# Inequities in the Hypertension and Diabetes Care Cascade: A Comparison of SES and Insurance in China, the US, and the UK

**DOI:** 10.3390/healthcare14040501

**Published:** 2026-02-15

**Authors:** Yutong Nie, Qiaorong Huang, Wentong Meng, Xue Li, Lei Chen, Xianming Mo

**Affiliations:** Laboratory of Stem Cell Biology, Frontiers Science Center for Disease-Related Molecular Network, Department of Neurology, West China Hospital, Sichuan University, Chengdu 610041, China; nieyt23@stu.scu.edu.cn (Y.N.);

**Keywords:** hypertension, diabetes, care cascade, socioeconomic status, health insurance, health equity, cross-national comparison

## Abstract

**Background/Objectives**: Socioeconomic status (SES) and health insurance are critical determinants of chronic disease outcomes. This study evaluates their impact on the hypertension and diabetes “care cascade” (diagnosis, treatment, and control) across three distinct health systems: China, the United States (US), and the United Kingdom (UK). **Methods**: We analyzed cross-sectional data from pooled survey waves of the China Health and Retirement Longitudinal Study (CHARLS), the US National Health and Nutrition Examination Survey (NHANES), and the English Longitudinal Study of Ageing (ELSA). The final analytic sample comprised a total of 46,054 participants with hypertension and 11,805 with diabetes. Logistic regression model was employed to estimate the associations of education, wealth, and health insurance with disease management outcomes. **Results**: Significant cross-national heterogeneity was observed. China exhibited the steepest attrition in the care cascade, with disparities strongly linked to insurance fragmentation; notably, Urban Employee Insurance was associated with significantly better outcomes compared to the Rural Cooperative Medical Scheme. In the US, health insurance was strongly associated with diagnosis and treatment initiation but showed attenuated associations with disease control, suggesting that financial barriers (“underinsurance”) may persist. The UK demonstrated the highest equity in access due to universal National Health Service coverage, though education remained a predictor for diabetes identification; moreover, a persistent wealth-based gradient in disease control remained despite universal access. **Conclusions**: Universal health coverage effectively mitigates access barriers but does not eliminate inequalities driven by cumulative socioeconomic disadvantage. Achieving equity requires context-specific strategies: reducing insurance fragmentation in China, minimizing out-of-pocket costs in the US, and addressing upstream social determinants in the UK.

## 1. Introduction

Hypertension and diabetes represent the leading contributors to the global burden of non-communicable diseases (NCDs) and are the primary risk factors for cardiovascular morbidity and mortality worldwide. The effective mitigation of these chronic conditions relies fundamentally on the continuity of care, conceptualized as the “care cascade”—a sequential continuum spanning screening, diagnosis, treatment initiation, and ultimately, disease control [1]. Despite the widespread availability of cost-effective pharmacological interventions, recent global epidemiological surveillance reveals a concerning stagnation in management efficiency. Large-scale analyses by the NCD Risk Factor Collaboration indicate that a substantial proportion of affected individuals remain undiagnosed or inadequately treated, resulting in suboptimal control rates across diverse healthcare settings [2,3]. Addressing these systemic gaps in the care cascade is, therefore, critical for reducing preventable disease burdens and achieving international health targets.

Crucially, deficits in the care cascade are not randomly distributed but are structured by distinct socioeconomic and insurance-related mechanisms that may differentially impact specific stages of care. While health insurance primarily functions as a financial “gateway”—facilitating access points such as diagnosis and treatment initiation—it does not guarantee the long-term maintenance required for disease control [4,5]. Achieving control often demands broader socioeconomic resources, where education and wealth may operate through divergent pathways. Education is hypothesized to drive health literacy, enhancing an individual’s cognitive capacity to navigate complex healthcare systems, communicate with providers, and adhere to treatment protocols [6]. Conversely, household wealth represents material resources, directly determining the affordability of copayments, healthy diets, and stress-free living environments essential for physiological regulation [7]. Therefore, mere coverage may mitigate access barriers, but inequities in health outcomes likely persist due to these entrenched socioeconomic disadvantages. Indeed, evidence suggests that even with insurance, higher-income groups often derive greater benefits from health services, leaving lower-SES patients vulnerable to catastrophic expenditures and compromised long-term adherence [8,9,10].

Existing comparative studies have predominantly relied on bilateral analyses, such as contrasts between China and the United States (US) [11] or between the US and the United Kingdom (UK) [12], often overlooking the unique insights afforded by a trilateral comparison of these distinct healthcare archetypes. These three nations represent divergent models of health financing and delivery that naturally predispose populations to varying health trajectories: China exemplifies a rapidly developing economy with a social insurance system undergoing comprehensive yet fragmented reform; the US represents a market-based model characterized by high expenditure but significant coverage gaps and a pronounced wealth-health gradient [12,13]; while the UK operates under a tax-funded, universal National Health Service (NHS) designed to minimize financial barriers to access [14]. Although socioeconomic disparities in cardiovascular risk factors have been documented within each setting [11,12,15], there remains a paucity of research utilizing rigorously harmonized data to systematically evaluate how these systemic differences modulate the association between socioeconomic status, health insurance, and the entire continuum of chronic disease management.

To bridge this knowledge gap, the present study integrates harmonized individual-level data from three nationally representative cohorts—the China Health and Retirement Longitudinal Study (CHARLS), the United States National Health and Nutrition Examination Survey (NHANES), and the English Longitudinal Study of Ageing (ELSA). To our knowledge, this is the first study to simultaneously disentangle these complex relationships across the full care cascade for both hypertension and diabetes within such a trilateral framework. Our primary objective is to systematically evaluate the differential associations of socioeconomic status and health insurance coverage with each stage of the chronic disease care cascade, encompassing diagnosis, treatment, and control. By elucidating how these associations vary across distinct health system architectures, this analysis seeks to uncover the mechanisms through which healthcare financing and delivery models may mitigate or exacerbate health inequalities, thereby providing empirical evidence to inform the design of more equitable global health policies.

## 2. Materials and Methods

### 2.1. Data Sources and Study Population

This study employed a comparative cross-sectional design using harmonized individual-level data from three nationally representative cohorts: the CHARLS, the NHANES, and ELSA. Detailed descriptions of the sampling design, data collection procedures, and biomarker protocols for each cohort are provided in Supplementary Materials Text S1.

China (CHARLS): We utilized data from Waves 1, 2, and 3 (collected in 2011, 2013, and 2015) of CHARLS. This survey covers residents across 28 provinces in China and provides longitudinal data on demographic and health-related characteristics [16].

The US (NHANES): We aggregated data from six continuous cycles of NHANES (2007–2008 through 2017–2018). NHANES is a cross-sectional survey assessing the health and nutritional status of the US civilian non-institutionalized population via household interviews and Mobile Examination Centers (MEC) (https://www.cdc.gov/nchs/nhanes/, accessed on 1 November 2025).

The UK (ELSA): We pooled data from four distinct waves of ELSA (Wave 2 [2004,2005], Wave 4 [2008,2009], Wave 6 [2012,2013], and Wave 8 [2016,2017]). These specific waves were selected because they included nurse assessment visits for biomarker collection [17].

**Figure 1:**
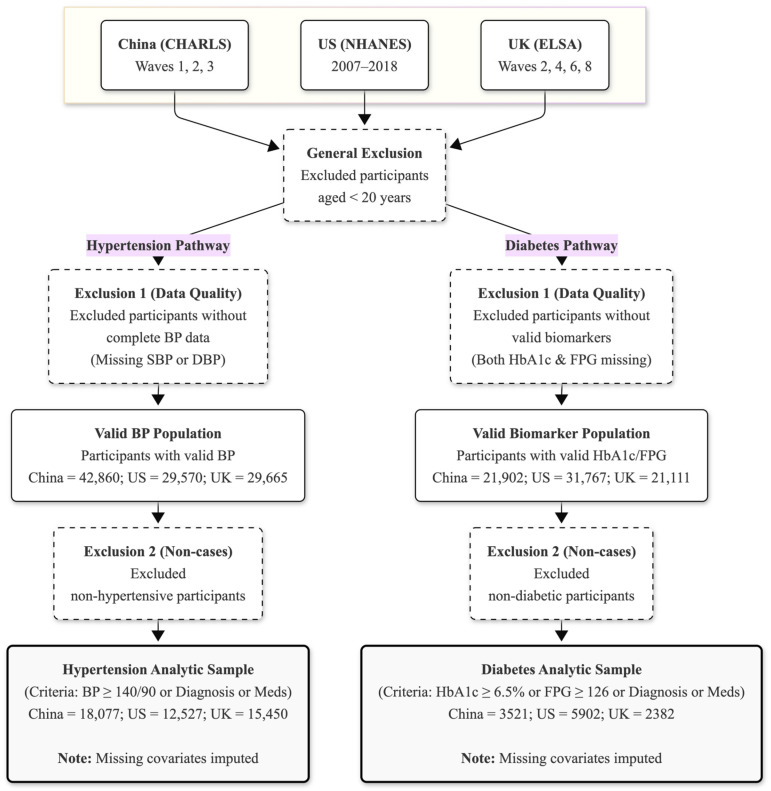
Flowchart of participant selection for the study. The diagram illustrates the derivation of the final analytic sample from three harmonized cohorts: the China Health and Retirement Longitudinal Study (CHARLS), the US National Health and Nutrition Examination Survey (NHANES), and the UK English Longitudinal Study of Ageing (ELSA). Abbreviations: US, the United States; UK, the United Kingdom; BP, blood pressure, SBP, systolic blood pressure; DBP, diastolic blood pressure; FPG, fasting plasma glucose; HbA1c, glycated hemoglobin.

To analyze nationally representative populations with distinct age structures, we included participants from all three cohorts. The study population comprises the general adult population in the US (NHANES), contrasted with the aging cohorts in China (CHARLS) and the UK (ELSA), which primarily target middle-aged and older adults. Specifically, individuals aged below 20 years were excluded. We further restricted the analytic sample to participants with valid measurements for physiological indicators necessary to define disease status (systolic/diastolic blood pressure [SBP/DBP] for hypertension; fasting plasma glucose [FPG] or glycated hemoglobin [HbA1c] for diabetes). Participants with missing values for key physiological variables defining the disease status (BP, HbA1c, or FPG) were excluded from the analytic cohort. In contrast, missing values for sociodemographic covariates were handled using multiple imputation, as detailed in the Statistical Analysis Section. The detailed participant selection process is presented in Figure 1.

### 2.2. Ethical Considerations

This study involves the secondary analysis of publicly available, de-identified datasets. The original surveys received ethical approval from their respective institutional review boards: the Biomedical Ethics Review Committee of Peking University (CHARLS), the National Center for Health Statistics Research Ethics Review Board (NHANES), and the London Multicentre Research Ethics Committee (ELSA). All participants provided written informed consent.

### 2.3. Disease Definitions and the Care Cascade

We defined the prevalence of hypertension and diabetes based on a combination of self-reported physician diagnosis, medication use, and physiological measurements. Hypertension was defined as having a mean SBP ≥ 140 mmHg, a mean DBP ≥ 90 mmHg, a self-reported diagnosis, or the current use of antihypertensive medication. Participants were classified as having diabetes if they reported a physician diagnosis, were currently using glucose-lowering medication or insulin, or met physiological criteria. Physiologically, HbA1c was prioritized: diabetes was defined as an HbA1c level ≥ 6.5%. For participants with missing HbA1c data, FPG was used as the secondary criterion, with a threshold of ≥ 126 mg/dL.

Based on these definitions, we constructed the “care cascade” comprising three sequential stages: (1) Diagnosis (Awareness): defined as having a self-reported professional diagnosis or current use of medication; (2) Treatment: defined as the current use of medication for specific condition; and (3) Control: defined as achieving clinical targets. For hypertension, control was defined as BP < 140/90 mmHg. For diabetes, glycemic control was defined as an HbA1c level < 7.0%, or alternatively as an FPG level < 126 mg/dL for participants for whom diabetes status was determined by FPG due to missing HbA1c data.

### 2.4. SES and Health Insurance

SES was evaluated using education and economic indicators. Education was harmonized across the three countries into three levels: Low (primary school or below/less than high school), medium (middle or high school/GED), and high (college/university degree or above). Economic status was assessed using country-specific indicators to capture relative socioeconomic standing: annual per capita household income for China, the Poverty Income Ratio (PIR) for the US, and total household wealth for the UK. To facilitate comparison, these continuous economic variables were categorized into quartiles (Q1–Q4) within each country. Health insurance coverage was categorized into country-specific groups to reflect the distinct financing structures and national characteristics of each healthcare system. China (CHARLS): Participants were classified into four groups: urban employee insurance, urban resident/other insurance, New Rural Cooperative Medical Scheme (NCMS), and uninsured. US (NHANES): Insurance status was categorized into private insurance, public insurance, and uninsured, with private coverage taking precedence in cases of dual enrollment. UK (ELSA): Given the universal coverage of the National Health Service (NHS), status was dichotomized into participants holding supplementary private insurance versus those relying solely on the NHS (NHS only).

Detailed classification definitions and coding logic for these insurance categories are provided in Supplementary Materials Table S1.

### 2.5. Covariates

To isolate the independent effects of SES and insurance, we adjusted for a comprehensive set of covariates in our multivariable models. These included demographic factors (age, sex) and race. Recognizing that race/ethnicity captures distinct social and structural dynamics in each setting, we applied country-specific categorizations: Han Chinese vs. minorities for China, White vs. Others for the UK, and specific racial/ethnic categories (Non-Hispanic White, Black, Mexican American, Other) for the US. To assess the extent to which socioeconomic disparities are mediated by behavioral and biological pathways, Model 2 further adjusted for potential mediators including smoking status, alcohol consumption, and body mass index (BMI), calculated as weight in kilograms divided by height in meters squared (kg/m^2^). Detailed definitions for these covariates are provided in Supplementary Materials Table S1.

### 2.6. Statistical Analysis

All statistical analyses were performed using R software (version 4.5.1). To ensure national representativeness and account for complex survey designs, appropriate sampling weights, clustering, and stratification variables were applied in all analyses. Specifically, we employed dataset-specific weighting strategies to ensure reproducibility and account for biomarker non-response: (1) For CHARLS, individual biomarker weights were applied to adjust for non-participation in blood collection; (2) for NHANES, to account for the aggregation of six survey cycles (2007–2018), we constructed a pooled analytic weight by dividing the 2-year Mobile Examination Center weight (WTMEC2YR) by six, in accordance with NCHS analytical guidelines; and (3) for ELSA, cross-sectional nurse weights were applied to correct for non-response to the nurse visit and blood collection components. Baseline characteristics were described using weighted means for continuous variables and weighted percentages for categorical variables.

**Figure 2:**
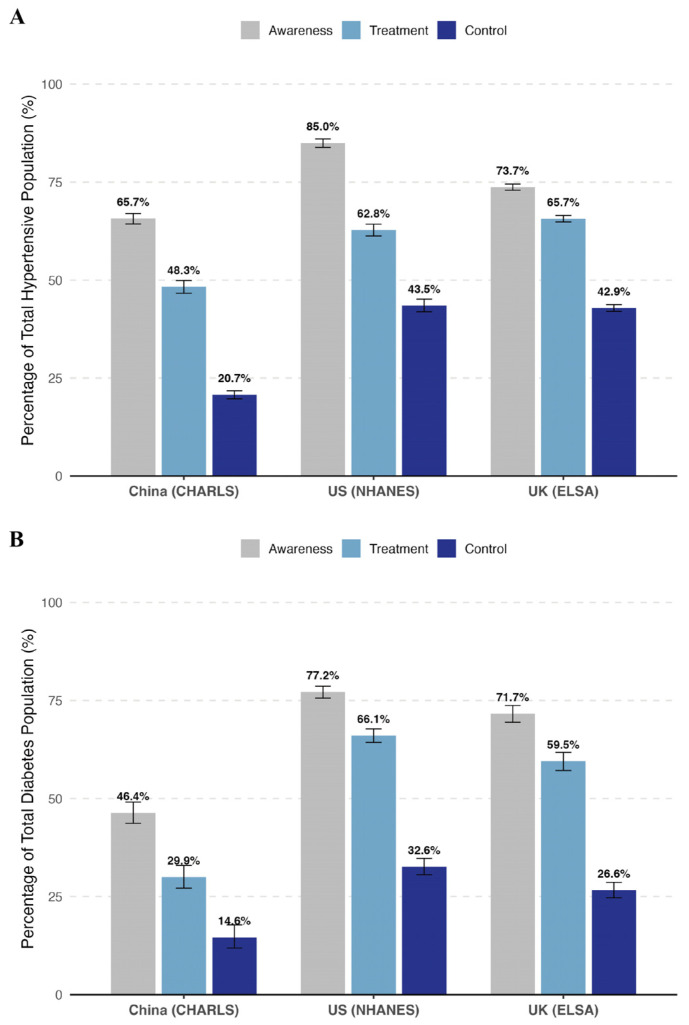
Care cascade for hypertension and diabetes among adult populations in China, the United States, and the United Kingdom. Bar charts display the weighted prevalence rates (95% confidence intervals) of the three stages of the care cascade: Diagnosis (Awareness), Treatment, and Control. (**A**) Hypertension: Diagnosis as self-reported history of professional diagnosis or current use of antihypertensive medication; Treatment as use of antihypertensive medication; Control as SBP < 140 mmHg and DBP < 90 mmHg among the total estimated hypertensive population. (**B**) Diabetes: Diagnosis as self-reported history of professional diagnosis or current use of insulin or oral hypoglycemic agents; Treatment as use of insulin or oral hypoglycemic agents; Control as HbA1c < 7.0% (or FPG < 126 mg/dL if HbA1c unavailable) among the total estimated diabetic population. All estimates were weighted to represent the national adult populations. Abbreviations: US, the United States; UK, the United Kingdom; BP, blood pressure; HbA1c, glycated hemoglobin; FPG, fasting plasma glucose.

To ensure accurate disease ascertainment, the study cohort was strictly defined by excluding participants with missing physiological indicators (BP, HbA1c, or FPG). The detailed step-by-step derivation of the analytic cohorts and exclusion rates by country are presented in Supplementary Materials Table S6. Based on this identified disease population, we utilized two distinct denominator approaches to assess the care cascade. For descriptive visualizations (Figure 2), a ‘fixed denominator’ approach was used, where proportions were calculated relative to the total estimated disease population. In contrast, for the multivariable logistic regression analyses, we employed a ‘conditional’ analytic sample approach. Crucially, for each regression stage, participants with missing survey responses regarding diagnosis or medication status were excluded (listwise deletion). Thus, the analytic sample for each model consisted solely of participants with valid binary outcomes (coded as 0 or 1), ensuring that the denominator represented the sum of valid responses.

We employed survey-weighted multivariable logistic regression models to estimate the associations of SES and health insurance with the odds of diagnosis, treatment, and control. In all analyses, the lowest socioeconomic groups and Uninsured/NHS-only groups served as reference categories. Data handling followed a unified protocol: (1) Cohort Identification: Missing values for physiological indicators were excluded to strictly define the disease population; (2) Outcome Variables: As described above, missing values for self-reported diagnosis and medication status were excluded from regression models to strictly define the conditional steps; and (3) Covariates: Prior to imputation, data quality checks were performed for continuous variables. Extreme outliers representing obvious coding errors were recoded as missing. Subsequently, missing values for all sociodemographic and lifestyle covariates were handled using Multiple Imputation by Chained Equations (MICE) to generate five imputed datasets, with results pooled using Rubin’s rules. To ensure robustness, we conducted four sets of sensitivity analyses: (1) using unweighted models; (2) complete-case analyses (without imputation); (3) restricting the sample to participants aged ≥50 years to address age structure differences; and (4) adjusting for survey years/waves to control for secular trends. Statistical significance was defined as a two-sided *p*-value < 0.05.

### 2.7. Declaration of Generative AI and AI-Assisted Technologies in the Manuscript Preparation Process

During the preparation of this work, the authors used deepseek in order to improve the readability and language quality of the manuscript. After using this tool, the authors reviewed and edited the content as needed and take full responsibility for the content of the published article.

## 3. Results

### 3.1. Study Population and Baseline Characteristics

From an initial pooled cohort of 196,497 individuals extracted from the CHARLS, NHANES, and ELSA databases, participants were sequentially excluded based on missing physiological data, including blood pressure measurements or diabetes biomarkers (HbA1c and FPG). Consequently, the final analytic samples comprised 18,077 (China), 12,527 (US), and 15,450 (UK) participants with hypertension, alongside 3521 (China), 5902 (US), and 2382 (UK) participants with diabetes.

The baseline characteristics of the included populations, summarized in Table 1, highlight substantial demographic and socioeconomic heterogeneity across the three cohorts. Demographically, the UK cohort was the oldest, with a mean age of approximately 69 years across both disease groups, whereas the US hypertension population was notably younger, with a mean age of 59.8 years. In terms of anthropometric indicators, a distinct gradient in adiposity was observed; the US cohort exhibited the highest burden of obesity, indicated by the highest mean BMI (Hypertension: 30.74 kg/m^2^; Diabetes: 32.50 kg/m^2^), while Chinese participants presented with the lowest mean BMI (~25 kg/m^2^). Socioeconomic disparities were equally pronounced, particularly regarding educational attainment. The Chinese cohort was predominantly characterized by lower education levels, with over 86% of hypertensive participants having completed primary education or below. Conversely, the US cohort demonstrated the highest educational attainment, with more than half of the hypertensive participants (56.2%) having attended college or university.

### 3.2. Cross-National Comparison of Care Cascades for Hypertension and Diabetes

The continuum of care for hypertension, illustrated in Figure 2A, revealed marked disparities in management indicators across the three cohorts, with the Chinese population experiencing the steepest attrition at every stage. Detailed step-by-step attrition rates, based on unweighted participant counts, are provided in Supplementary Materials Table S7 to quantify the ‘leakage’ at each stage. In terms of diagnosis, the US cohort demonstrated the highest level of awareness (85.0%), significantly outpacing both the UK (73.7%) and China (65.7%). While the US led in detection, the UK achieved the highest treatment rate among the total hypertensive population (65.7%), slightly surpassing the US (62.8%). In stark contrast, treatment coverage in China was considerably lower, extending to less than half (48.3%) of the estimated hypertensive population. This cumulative loss of patients along the cascade corresponded to profound inequalities in final health outcomes: blood pressure control rates in the US (43.5%) and UK (42.9%) were more than double that of China, where only 20.7% of participants achieved therapeutic targets.

A similar, albeit more pronounced, pattern of substantial unmet need was observed in the diabetes care cascade (Figure 2B). Awareness gaps were particularly acute in the Chinese cohort, where less than half (46.4%) of individuals with diabetes had been diagnosed, compared to over 70% in both Western cohorts (US: 77.2%; UK: 71.7%). This initial deficit persisted through subsequent stages, coinciding with significantly lower treatment coverage in China (29.9%) relative to the US (66.1%) and UK (59.5%). Consequently, effective glycemic control remained a global challenge, achieved by a minority of the population across all settings. However, the deficit was most severe in China, with a control rate of merely 14.6%, whereas the US and UK maintained comparatively higher control rates of 32.6% and 26.6%, respectively. Overall, the US cohort generally exhibited the most robust retention across the care continuum for diabetes, while the UK showed a notable drop-off between treatment and control stages.

### 3.3. Association of Socioeconomic Factors with Disease Management

**Figure 3:**
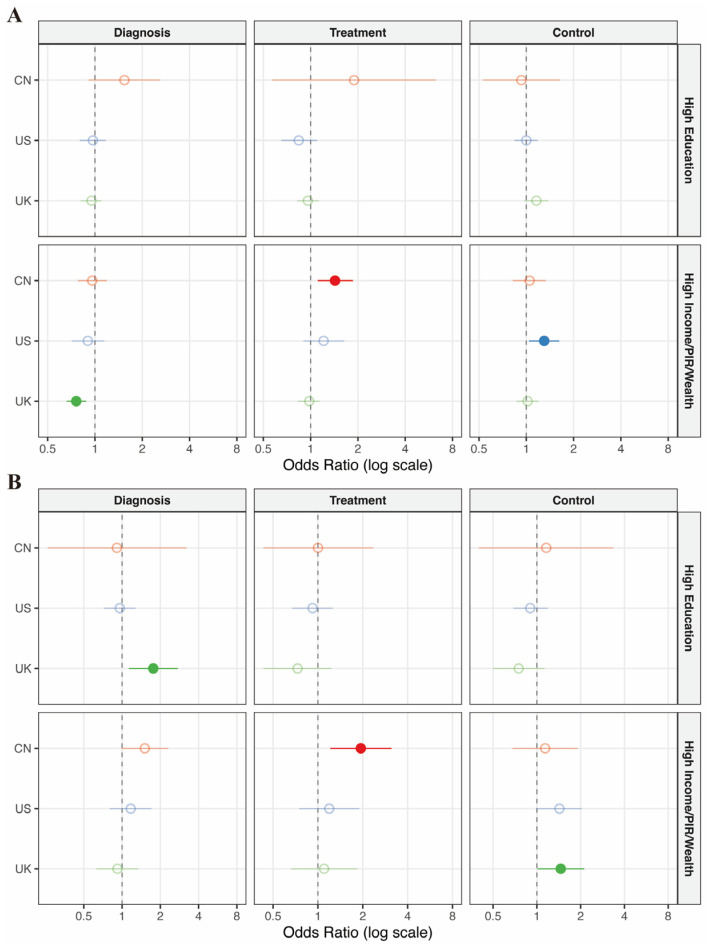
Association between socioeconomic status and the care cascade for hypertension and diabetes across China, the United States, and the United Kingdom. Forest plots display the multivariable-adjusted Odds Ratios (ORs) and 95% Confidence Intervals (CIs) for (**A**) Hypertension and (**B**) Diabetes. The vertical grey dotted line at Odds Ratio (OR) = 1 represents the line of no effect. Colors indicate different cohorts: red for China, blue for the US, and green for the UK. Solid circles indicate statistically significant associations (*p* < 0.05), while hollow circles indicate non-significant associations. The models were adjusted for age, gender, race, smoking status, alcohol consumption, and BMI. Variables: High Education: Defined as having a university degree or above (Reference: Primary school or below). High Income/PIR/Wealth: Defined as the top quartile of household income or wealth (Reference: Bottom quartile). Abbreviations: CN, China; US, the United States; UK, the United Kingdom; PIR, poverty income ratio.

**Figure 4:**
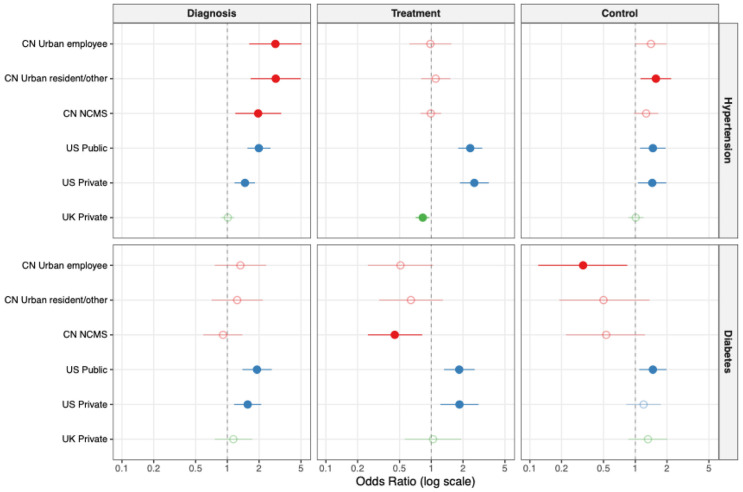
Impact of health insurance schemes on the management of hypertension and diabetes in China, the United States, and the United Kingdom. The forest plot illustrates the multivariable-adjusted Odds Ratios (ORs) and 95% Confidence Intervals (CIs) for the association between specific health insurance types and disease management outcomes (Diagnosis, Treatment, and Control). The top panel displays results for Hypertension, and the bottom panel displays results for Diabetes, as labeled on the right side of the plot. Colors indicate different country cohorts: red for China (CN), blue for the United States (US), and green for the United Kingdom (UK). Solid circles indicate statistically significant associations (*p* < 0.05), while hollow circles indicate non-significant associations. The vertical grey dotted line at OR = 1 represents the line of no effect. Reference Groups: China: Participants with No Insurance served as the reference group. Comparisons include Urban Employee Basic Medical Insurance, Urban Resident Basic Medical Insurance, and New Cooperative Medical Scheme (NCMS). United States: Participants with No Insurance served as the reference group. Comparisons include Public Insurance and Private Insurance. United Kingdom: Participants with National Health Service coverage only served as the reference group. The plot displays the additional effect of having Private Health Insurance. Models were adjusted for age, gender, race, smoking status, alcohol consumption and, BMI. Abbreviations: CN, China; US, the United States; UK, the United Kingdom; NCMS, New Cooperative Medical Scheme.

To evaluate the associations of socioeconomic determinants with the care cascade, we performed multivariable logistic regression analyses, adjusting for demographic factors (Model 1) and further controlling for lifestyle risk factors including smoking status, alcohol consumption, and BMI (Model 2). Detailed numerical results for all associations are presented in Table 2, Figure 3 and Figure 4, and Supplementary Materials Table S2.

Beyond insurance, socioeconomic and educational attainment—visualized in Figure 3—demonstrated heterogeneous associations with disease management across the three countries. Wealth disparities were most evident in hypertension management: high income was significantly associated with improved treatment adherence in China (AOR 1.43, 95% CI 1.11–1.86) and better blood pressure control in the US (AOR 1.3, 95% CI 1.04–1.62). Conversely, within the UK cohort, higher wealth was inversely associated with hypertension diagnosis (AOR 0.76, 95% CI 0.66–0.88). This finding implies a complex relationship that requires cautious interpretation; it may reflect lower engagement with primary care screening among wealthier groups (the ‘healthy worker effect’) or, as indicated by our sensitivity analysis adjusting for survey years (Table S5), temporal shifts in screening intensity across ELSA waves. As shown in Figure 3, educational attainment appeared to play a more limited role compared to financial indicators; its impact was largely statistically insignificant across most outcomes, with the notable exception of diabetes diagnosis in the UK, where higher education was strongly associated with a greater likelihood of being diagnosed (AOR 1.76, 95% CI 1.13–2.75). These findings underscore that while financial barriers (income and insurance) are the predominant constraints in the US and China, the UK’s barriers may be more related to health literacy or system engagement patterns among specific socioeconomic strata.

Health insurance coverage emerged as a pivotal factor associated with of care continuity, particularly within the Chinese and US cohorts, a trend visibly illustrated in Figure 4. In China, participants covered by Employee or Urban Resident insurance schemes exhibited a more than two-fold increase in the odds of hypertension diagnosis compared to the uninsured (e.g., Employee Insurance: AOR 2.86, 95% CI 1.61–5.05), with NCMS also showing a positive, albeit weaker, association. Similarly, in the US, possession of either public or private insurance was consistently associated with significantly better outcomes across the entire hypertension cascade, substantially increasing the likelihood of diagnosis, treatment, and control relative to the uninsured. However, the positive association of insurance was not universal; notably, in the Chinese diabetes cohort, enrollment in NCMS was paradoxically associated with lower odds of receiving treatment (AOR 0.45, 95% CI 0.25–0.82). In the UK context, holding private insurance (versus NHS only) was associated with lower odds of hypertension treatment (AOR 0.83, 95% CI 0.71–0.96), suggesting a distinct utilization pattern in the British dual-system healthcare landscape.

### 3.4. Sensitivity Analyses

To ensure the robustness of our findings and address potential biases arising from missing data, differing age structures, or secular trends, we conducted four sets of sensitivity analyses: (1) restricting the sample to participants aged ≥ 50 years; (2) adjusting for survey years/waves; (3) using complete-case data (no imputation); and (4) using unweighted models.

First, age-restricted analyses (aged ≥ 50 years; Supplementary Materials Table S4) largely confirmed the primary findings, particularly the strong associations between health insurance and access to care in the US and China. While the direction of associations remained consistent, statistical significance was attenuated for specific subgroups (e.g., private insurance and diabetes diagnosis in the US) likely due to reduced statistical power. Notably, the wealth gradient for diabetes control—evident in the full sample—was substantially weaker in this older cohort, suggesting that the influence of material resources on clinical control may diminish with age compared to the general adult population.

Second, regarding secular trends (Supplementary Materials Table S5), adjusting for survey years had minimal impact on the primary associations between insurance/income and disease access. However, temporal adjustment did clarify certain specific findings: the inverse association between wealth and hypertension diagnosis in the UK became non-significant, confirming that the initial observation was likely driven by temporal shifts in screening intensity. Similarly, the attenuation of certain control-related gradients (e.g., in China and the US) suggests that secular improvements in clinical management partially confounded these specific associations in the unadjusted models.

Finally, analyses utilizing unweighted and complete-case data (Supplementary Materials Table S3) demonstrated high consistency with the primary weighted, multiple-imputation models regarding the direction and magnitude of effect estimates. Most primary associations, particularly those regarding health insurance, remained statistically significant. However, statistical significance was attenuated for specific socioeconomic indicators (e.g., education and hypertension diagnosis in the US; income and diabetes treatment in the UK). This attenuation likely reflects the reduced statistical power from listwise deletion in complete-case analysis and the loss of population representativeness when sampling weights are excluded.

## 4. Discussion

To the best of our knowledge, this study represents the first trilateral comparison utilizing harmonized, nationally representative data to disentangle the complex associations between socioeconomic status, health insurance, and the complete care cascade for hypertension and diabetes across China, the US, and the UK. Our findings reveal a pervasive socioeconomic gradient in disease management across all three nations, yet the magnitude and mechanisms of these disparities exhibit profound heterogeneity contingent upon the underlying healthcare system. China displayed the steepest socioeconomic hierarchy, where disparities were heavily driven by the fragmentation of health insurance schemes; notably, individuals covered by the Urban Employee Basic Medical Insurance consistently outperformed those under the NCMS or the uninsured, particularly in the diagnosis and treatment stages. In the US, health insurance functioned primarily as a critical “gateway” to the healthcare system, exerting a substantial influence on diagnosis and treatment initiation—the entry points of the cascade—while its association with effective disease control was comparatively attenuated, suggesting that access alone does not guarantee quality of care. Conversely, the UK demonstrated the highest degree of equity across the care cascade, likely attributable to the universal coverage provided by the NHS; nevertheless, a persistent wealth-based gradient in health outcomes remains evident, indicating that removing financial barriers at the point of service cannot fully neutralize the broader health impacts of cumulative economic disadvantage.

A notable finding across our stratified models was the divergent roles of education and financial indicators. While household wealth and insurance coverage were consistently strong predictors of disease management, the independent association with educational attainment was largely attenuated in fully adjusted models. We hypothesize several mechanisms for this pattern. First, for the management of established chronic conditions like hypertension and diabetes, material resources (the ability to afford continuous medication, healthy dietary options, and transportation) may be more proximal and decisive determinants of adherence than health literacy (often proxied by education). Second, measurement limitations may play a role: in older cohorts—particularly in developing contexts like China—educational attainment reflects opportunities available decades ago and may be a less sensitive indicator of current socioeconomic agency than accumulated wealth. Thus, while education provides the cognitive foundation for health behaviors, our results suggest that without the requisite financial means (insurance and wealth) to execute these behaviors, high health literacy alone may not translate into improved clinical control.

The pronounced socioeconomic gradient observed in China likely reflects the systemic fragmentation characterizing its healthcare system during a period of rapid epidemiological transition. While China has achieved impressive strides toward universal health coverage, the depth of financial protection and access to quality care remain heavily stratified by occupational and residential status [18]. Our analysis indicates that beneficiaries of the Urban Employee Basic Medical Insurance consistently achieved better access to diagnosis and treatment than those covered by NCMS, a finding that underscores the persistent regional and sectoral segmentation of the Chinese health insurance landscape [18]. This disparity suggests that for the rural and lower-income population, health insurance functions more as a safety net for catastrophic inpatient events rather than a facilitator of routine chronic disease management. The structural deficits in the primary healthcare system further exacerbate these inequalities; despite widespread infrastructure investment, primary care providers in resource-constrained areas often face challenges related to workforce qualifications, disjointed health information systems, and financial incentives that do not effectively encourage longitudinal risk factor control [19]. Consequently, effective disease management remains largely dependent on individual ability to pay, leaving the growing number of older adults in rural areas particularly vulnerable to adverse health events [20]. Given that population aging and metabolic risk factors are now the predominant drivers of the cardiovascular disease burden in China [5], bridging the quality gap between urban-employee and rural-resident schemes is an urgent priority for reducing national health inequities.

In the United States, our results illuminate the distinct function of health insurance as a necessary but insufficient determinant of optimal disease outcomes. While insurance coverage—particularly private plans—served as a potent facilitator for diagnosis and treatment initiation, its association with effective disease control was notably attenuated compared to the access phase. This divergence likely reflects the pervasive phenomenon of “underinsurance,” exacerbated by the proliferation of High-Deductible Health Plans (HDHPs) [21,22]. While HDHPs may lower monthly premiums, they impose substantial upfront costs that create financial barriers to medication adherence even among the insured population [23]. Recent evidence from a large national cohort indicates that HDHP enrollment is associated with statistically significant declines in the receipt of guideline-concordant care, including a 9.0 percentage-point reduction in adherence to recommended medications for chronic conditions [22]. Similarly, a systematic review found that HDHPs often reduce high-value diabetes monitoring and medication adherence, disproportionately affecting lower-income patients [21]. Our findings resonate with national surveillance data indicating that cost-related medication non-adherence (CRN) remains prevalent among U.S. adults with diabetes and cardiovascular conditions, particularly those relying on insulin or multiple pharmacotherapies [24,25]. Although the Affordable Care Act expanded coverage, the structure of many benefit packages often fails to shield patients from the cumulative costs of chronic disease management. Consequently, financial toxicity acts as a latent disruptor of continuity of care; patients may gain entry to the healthcare system but are subsequently forced to ration life-saving medications due to out-of-pocket costs [25]. This mechanism is further corroborated by evidence linking CRN to increased all-cause and disease-specific mortality in hypertensive and diabetic populations, underscoring that insurance expansion must be coupled with benefit designs that exempt high-value chronic disease medications from deductibles to translate access into tangible health gains [26,27].

The United Kingdom results demonstrate the relative success of the National Health Service (NHS) in minimizing access-related disparities, yet they simultaneously reveal the limitations of a healthcare-centric approach to health equity. While the universal coverage model effectively blunted the socioeconomic gradient in diagnosis and treatment initiation compared to the US and China, a persistent wealth-based hierarchy in disease control suggests that removing point-of-service financial barriers does not equate to neutralizing the cumulative biological impact of socioeconomic disadvantage [28]. Our findings align with longitudinal analyses of the English population indicating that wealth remains a robust predictor of health decline and multimorbidity onset in later life, independent of healthcare access [28]. It is important to note that, unlike in the US, private health insurance in the UK functions primarily as “supplementary” coverage for elective procedures and faster specialist access, rather than a prerequisite for primary chronic disease management which is universally covered by the NHS. Consequently, any observed associations with private insurance in the UK likely reflect selection biases (e.g., the “healthy worker effect” or higher baseline health status among the insured) rather than fundamental advantages in access to essential hypertension or diabetes care. This residual inequality highlights that the determinants of effective chronic disease management extend beyond clinical encounters to broader social determinants of health (SDOH). Specific upstream factors unique to the UK context likely drive these disparities. For instance, “fuel poverty”—the inability to afford adequate heating—remains a critical determinant of cardiovascular health in the UK; living in cold, damp housing has been directly linked to elevated blood pressure and respiratory morbidity among older adults [29]. Similarly, food insecurity and the high cost of healthy diets pose significant barriers to glycemic control for lower-income diabetic patients, necessitating interventions that go beyond medication to include social prescribing of healthy foods [30]. Furthermore, evidence from rapid reviews of supported housing in England underscores that poor-quality housing environments exacerbate health inequalities and mental wellbeing issues, creating a cycle of disadvantage that clinical care alone cannot break [31]. Although policy frameworks in England have explicitly targeted health inequalities, structural complexities within the NHS can still create “invisible barriers” for deprived populations; for instance, patients from lower socioeconomic backgrounds face distinct challenges related to appointment navigation, transport, and communication literacy that impede consistent engagement with outpatient services [32,33]. Furthermore, while population-wide interventions like the NHS Health Check programme have contributed to narrowing gaps in cardiovascular risk detection, their absolute impact is often constrained by suboptimal uptake and follow-through among the most vulnerable groups [34]. Thus, achieving true equity in outcomes requires integrating clinical care with broader social support mechanisms that address these upstream drivers of ill health. We also noted that the inverse association between wealth and hypertension diagnosis observed in the unadjusted UK model was attenuated after controlling for survey waves. This suggests that the initial finding may have been an artifact of temporal shifts in screening intensity across the study period rather than a direct biological effect.

Our study has several limitations. First, the cross-sectional design precludes causal inferences regarding the impact of socioeconomic factors on disease management. Second, while we applied a uniform inclusion criterion excluding individuals aged below 20 years, the inherent age structures of the source populations differ. Specifically, NHANES targets the general US population, whereas CHARLS and ELSA track aging populations. However, we conducted robust sensitivity analyses restricted to participants aged ≥50 years (Supplementary Table S4), which confirmed that our core findings regarding financial barriers remain consistent across comparable age groups. Third, data were collected at slightly different time points (2011–2015 for CHARLS vs. pooled cycles for NHANES and ELSA); we addressed this by adjusting for survey waves in our sensitivity analysis (Supplementary Table S5), which largely supported the pooled estimates. Fourth, we acknowledge significant challenges in cross-national measurement equivalence. Socioeconomic indicators—particularly education—may not be fully equivalent across diverse cultural and historical contexts. For instance, “primary education” in rural China represents a different relative social standing and human capital implication compared to the US or UK. Similarly, the construct of “race/ethnicity” captures distinct structural dynamics in each setting, precluding direct equivalence. We also note that the inclusion of lifestyle factors (e.g., BMI) in Model 2 may represent “over-adjustment” if these factors act as mediators; however, this allows us to estimate the proportion of the gradient attributable to modifiable risks. Finally, subtle differences in questionnaire wording or laboratory assay methods across countries could contribute to residual variations. Consequently, given these inherent structural and methodological heterogeneities, our findings should be interpreted as an evaluation of how socioeconomic mechanisms operate within each distinct system, rather than as a normative ranking of healthcare system performance.

## 5. Conclusions

In conclusion, subject to the limitations of the cross-sectional design, this trilateral comparison demonstrates that while universal health coverage—as exemplified by the UK—substantially attenuates access-related disparities in the care cascade, it does not entirely eliminate the health gradient driven by cumulative socioeconomic disadvantage. Our findings suggest that achieving health equity requires context-specific policy interventions. For China, the urgent priority lies in reducing the fragmentation between employee and resident insurance schemes and strengthening primary care capacity in rural areas to ensure that high coverage rates translate into effective disease control. For the US, policy efforts must focus on closing coverage gaps and redesigning insurance benefits—specifically regarding high-deductible plans—to lower out-of-pocket costs, thereby preventing financial toxicity from compromising treatment adherence. For the UK, the persistence of wealth-based inequalities within a universal system suggests that future interventions must extend beyond the clinical setting to address the upstream social determinants of health. Ultimately, realizing the promise of global health equity demands a synergistic approach that combines robust financial protection with high-quality, accessible, and integrated primary care delivery.

## Figures and Tables

**Table 1:** Baseline characteristics of participants with hypertension and diabetes in China, the US, and the UK.

Variables	Hypertension Population China (*n* = 18,077)	US (*n* = 12,527)	UK (*n* = 15,450)	Diabetes Population China (*n* = 3521)	US (*n* = 5902)	UK (*n* = 2382)
Demographics						
Age, years, mean (SD)	62.8 (10.5)	59.8 (15.1)	69.0 (10.3)	61.8 (9.6)	61.1 (13.5)	69.1 (9.8)
Female, *n* (%)	9609 (51.9)	6216 (49.7)	8276 (51.9)	1976 (53.7)	2832 (48.0)	1241 (51.5)
Race/Ethnicity, *n* (%)						
Han Chinese	16,483 (91.8)	-	-	3252 (94.2)	-	-
Non-Hispanic White	-	5251 (68.3)	-	-	2012 (34.1)	-
Non-Hispanic Black	-	3314 (13.8)	-	-	1484 (25.1)	-
Mexican American/Hispanic	-	1529 (6.1)	-	-	1051 (17.8)	-
Other/Multi-Racial	-	2433 (11.8)	-	-	1355 (22.9)	-
White	-	-	15,056 (95.9)	-	-	2268 (91.5)
Education Level, *n* (%)						
Primary or below	16,307 (86.4)	3541 (18.4)	5969 (43.2)	3172 (85.3)	1999 (33.9)	969 (47.4)
Middle/High School	1479 (10.5)	3049 (25.4)	7279 (44.2)	279 (11.1)	1362 (23.1)	1112 (42.2)
College or above	291 (3.1)	5920 (56.2)	2202 (12.5)	70 (3.6)	2529 (42.9)	301 (10.4)
Economic Indicators						
Household Income (China), CNY, mean (SD)	23,608 (71,909)	-	-	21,549 (38,507)	-	-
Family Income PIR (US), mean (SD)	-	2.4 (1.6)	-	-	2.3 (1.5)	-
Household Wealth (UK), GBP, mean (SD)	-	-	298,197 (434,333)	-	-	252,737 (442,698)
Insurance Status, *n* (%)						
Urban Employee Insurance	2434 (20.8)	-	-	505 (23.3)	-	-
Urban Resident/Other	1730 (11.2)	-	-	368 (13.1)	-	-
NCMS	12,784 (61.1)	-	-	2420 (58.0)	-	-
Public Insurance	-	4318 (26.2)	-	-	2206 (37.5)	-
Private Insurance	-	6381 (62.1)	-	-	2812 (47.8)	-
Private Insurance	-	-	1817 (11.6)	-	-	216 (8.9)
Uninsured/NHS only	1082 (6.9)	1789 (11.7)	13,633 (88.4)	210 (5.6)	860 (14.6)	2166 (91.1)
Lifestyle						
Smoker, *n* (%)	7593 (42.0)	6192 (49.8)	9826 (64.0)	1413 (41.7)	2915 (49.4)	1599 (66.3)
Alcohol Consumer, *n* (%)	7946 (44.1)	8016 (50.2)	13,325 (84.8)	1467 (43.1)	3147 (62.9)	1909 (77.6)
BMI, kg/m^2^, mean (SD)	24.6 (4.8)	30.7 (7.1)	29.0 (5.3)	25.1 (5.8)	32.5 (7.6)	30.9 (5.9)
Clinical Characteristics						
SBP, mmHg, mean (SD)	146.2 (19.2)	135.6 (19.0)	141.3 (18.0)	-	-	-
DBP, mmHg, mean (SD)	82.9 (11.9)	72.2 (14.5)	76.4 (12.2)	-	-	-
FPG, mg/dL, mean (SD)	-	-	-	144.5 (58.4)	155.9 (61.8)	132.5 (47.9)
HbA1c, %, mean (SD)	-	-	-	6.8 (1.7)	7.3 (1.7)	7.2 (1.3)

Data presentation: Sample sizes (N) are presented as unweighted counts, while means (standard deviation, SD) and percentages (%) are weighted estimates. Abbreviations: US, the United States; UK, the United Kingdom; BMI, body mass index; SBP, systolic blood pressure; DBP, diastolic blood pressure; FPG, fasting plasma glucose; HbA1c, glycated hemoglobin; PIR, poverty income ratio; CNY, Chinese Yuan; GBP, Great British Pound; NCMS, New Rural Cooperative Medical Scheme; -, not applicable. Race/Ethnicity: Classifications are specific to each country’s dataset (Han for China; specific categories for the US; White for the UK). Economic Indicators: Economic status is measured by annual household income in China, PIR in the US, and total household wealth in the UK.

**Table 2:** Multivariable-adjusted associations of socioeconomic status and health insurance with the diagnosis, treatment, and control of hypertension and diabetes in China, the US, and the UK.

Variables	Hypertension Diagnosis AOR (95% CI)	Treatment AOR (95% CI)	Control AOR (95% CI)	Diabetes Diagnosis AOR (95% CI)	Treatment AOR (95% CI)	Control AOR (95% CI)
China						
Education Level						
Primary or below (Ref)	-	-	-	-	-	-
Middle/High School	1.01 (0.77, 1.32)	1.08 (0.82, 1.42)	1 (0.77, 1.28)	0.8 (0.51, 1.23)	0.84 (0.49, 1.45)	1.68 (0.78, 3.63)
College or above	1.54 (0.91, 2.6)	1.89 (0.57, 6.26)	0.93 (0.53, 1.64)	0.91 (0.26, 3.2)	1 (0.43, 2.35)	1.16 (0.4, 3.36)
Household Income						
Quartile 1 (Ref)	-	-	-	-	-	-
Quartile 2	**1.22 (1.01, 1.47)**	1.12 (0.96, 1.3)	0.99 (0.84, 1.16)	0.94 (0.66, 1.32)	1.03 (0.73, 1.46)	1.13 (0.71, 1.79)
Quartile 3	0.93 (0.69, 1.24)	1.14 (0.92, 1.43)	0.97 (0.79, 1.2)	0.99 (0.67, 1.44)	1.23 (0.82, 1.83)	1.43 (0.91, 2.25)
Quartile 4	0.96 (0.78, 1.19)	**1.43 (1.11, 1.86)**	1.05 (0.82, 1.33)	1.51 (0.99, 2.31)	**1.94 (1.21, 3.12)**	1.14 (0.68, 1.91)
Health Insurance						
Uninsured (Ref)	-	-	-	-	-	-
Urban Employee	**2.86 (1.61, 5.05)**	0.98 (0.62, 1.55)	1.41 (1, 1.99)	1.33 (0.76, 2.34)	0.51 (0.25, 1.04)	**0.32 (0.12, 0.84)**
Urban Resident/Other	**2.88 (1.66, 4.97)**	1.1 (0.8, 1.52)	**1.57 (1.12, 2.19)**	1.24 (0.71, 2.17)	0.64 (0.32, 1.29)	0.5 (0.19, 1.37)
NCMS	**1.96 (1.19, 3.25)**	0.99 (0.79, 1.24)	1.27 (0.97, 1.65)	0.91 (0.59, 1.39)	**0.45 (0.25, 0.82)**	0.53 (0.22, 1.24)
US						
Education Level						
Primary or below (Ref)	-	-	-	-	-	-
Middle/High School	**0.78 (0.65, 0.94)**	0.92 (0.72, 1.18)	1 (0.84, 1.2)	0.94 (0.71, 1.24)	0.91 (0.6, 1.38)	0.8 (0.63, 1.02)
College or above	0.97 (0.8, 1.17)	0.84 (0.65, 1.1)	1 (0.84, 1.18)	0.96 (0.72, 1.28)	0.92 (0.67, 1.26)	0.9 (0.69, 1.19)
PIR						
Quartile 1 (Ref)	-	-	-	-	-	-
Quartile 2	1.05 (0.87, 1.27)	0.93 (0.73, 1.19)	0.96 (0.81, 1.14)	1.11 (0.83, 1.48)	1.3 (0.94, 1.8)	**1.34 (1.04, 1.74)**
Quartile 3	1.08 (0.9, 1.28)	1.15 (0.88, 1.51)	**1.32 (1.07, 1.62)**	1.12 (0.84, 1.5)	1.17 (0.74, 1.86)	1.25 (0.92, 1.7)
Quartile 4	0.9 (0.71, 1.15)	1.21 (0.9, 1.64)	**1.3 (1.04, 1.62)**	1.17 (0.8, 1.7)	1.19 (0.75, 1.89)	**1.43 (1, 2.04)**
Health Insurance						
Uninsured (Ref)	-	-	-	-	-	-
Public Insurance	**1.99 (1.55, 2.57)**	**2.34 (1.8, 3.05)**	**1.47 (1.11, 1.94)**	**1.91 (1.39, 2.64)**	**1.84 (1.32, 2.58)**	**1.47 (1.09, 1.98)**
Private Insurance	**1.47 (1.17, 1.83)**	**2.56 (1.87, 3.52)**	**1.45 (1.06, 1.97)**	**1.56 (1.16, 2.1)**	**1.85 (1.22, 2.81)**	1.2 (0.82, 1.75)
UK						
Education Level						
Primary or below (Ref)	-	-	-	-	-	-
Middle/High School	0.97 (0.88, 1.08)	1.05 (0.93, 1.17)	1.1 (0.97, 1.23)	1.1 (0.85, 1.42)	**0.66 (0.47, 0.95**)	1.06 (0.81, 1.4)
College or above	0.95 (0.81, 1.1)	0.96 (0.82, 1.13)	1.16 (0.97, 1.38)	**1.76 (1.13, 2.75)**	0.73 (0.43, 1.23)	0.75 (0.5, 1.13)
Household Wealth						
Quartile 1 (Ref)	-	-	-	-	-	-
Quartile 2	**0.85 (0.73, 0.97)**	**0.86 (0.74, 1)**	0.9 (0.77, 1.05)	1.28 (0.89, 1.83)	1.36 (0.8, 2.31)	1.39 (0.97, 1.98)
Quartile 3	**0.83 (0.72, 0.96)**	0.95 (0.81, 1.11)	0.94 (0.8, 1.09)	1.08 (0.75, 1.57)	1.36 (0.82, 2.27)	1.33 (0.92, 1.93)
Quartile 4	**0.76 (0.66, 0.88)**	0.98 (0.83, 1.14)	1.02 (0.87, 1.2)	0.92 (0.63, 1.34)	1.1 (0.66, 1.84)	**1.46 (1.01, 2.12)**
Private Insurance						
No (Public Only) (Ref)	-	-	-	-	-	-
Yes	1.01 (0.87, 1.16)	**0.83 (0.71, 0.96)**	1.01 (0.86, 1.2)	1.14 (0.76, 1.72)	1.04 (0.56, 1.92)	1.32 (0.86, 2.02)

Data are presented as adjusted odds ratios (AOR) with 95% confidence intervals (CI). Bold values indicate statistical significance (*p* < 0.05). Models: Results are derived from Model 2, which is adjusted for age, sex, race, smoking status, alcohol consumption, and BMI. Reference Groups: The reference group (Ref) denotes the baseline category for comparison: the lowest education level (Primary/Below), the lowest income/wealth quartile (Q1), and “Uninsured” (for China/the US) or “Public Insurance Only” (for the UK). Abbreviations: US, the United States; UK, the United Kingdom; Ref, reference group; NCMS, New Cooperative Medical Scheme; PIR, poverty income ratio.

## Data Availability

The data analyzed in this study were obtained from publicly available datasets. The China Health and Retirement Longitudinal Study (CHARLS) data are available at http://charls.pku.edu.cn/ (accessed on 1 November 2025). The National Health and Nutrition Examination Survey (NHANES) data are available at https://wwwn.cdc.gov/nchs/nhanes/ (accessed on 1 November 2025). The English Longitudinal Study of Ageing (ELSA) data are available at https://beta.ukdataservice.ac.uk/datacatalogue/series/series?id=200011 (accessed on 1 November 2025).

## Supplementary Materials

The following supporting information can be downloaded at: https://www.mdpi.com/article/10.3390/healthcare14040501/s1, Text S1. Detailed Survey Methodology; S1.1. China (CHARLS); S1.2. United States (NHANES); S1.3. United Kingdom (ELSA); Table S1. Variable harmonization strategy and definitions across China (CHARLS), the US (NHANES), and the UK (ELSA); Table S2. Full multivariable logistic regression results for the association between socioeconomic status, covariates, and chronic disease management (Model 1 and Model 2); Table S3. Sensitivity analysis of the associations between socioeconomic status and disease management using complete-case data (weighted and unweighted models); Table S4. Sensitivity analysis of the associations between socioeconomic status and disease management restricted to participants aged ≥50 years; Table S5. Sensitivity analysis of the associations between socioeconomic status and disease management adjusting for survey years/waves to control for secular trends; Table S6. Derivation of analytic cohorts and participant exclusion flow due to missing physiological indicators; Table S7. Step-by-step sample attrition and unweighted participant counts across the hypertension and diabetes care cascades [16,17,35,36,37,38,39].

## Abbreviations

AOR	Adjusted Odds Ratio
CI	Confidence Interval
Ref	Reference group
SD	Standard Deviation
US	The United States
UK	The United Kingdom
BMI	Body Mass Index
BP	Blood Pressure
DBP	Diastolic Blood Pressure
SBP	Systolic Blood Pressure
FPG	Fasting Plasma Glucose
HbA1c	Glycated Hemoglobin
CRN	Cost-Related Medication Non-adherence
SES	Socioeconomic Status
CHARLS	China Health and Retirement Longitudinal Study
ELSA	English Longitudinal Study of Ageing
NHANES	National Health and Nutrition Examination Survey
GED	General Educational Development
MEC	Mobile Examination Center
NCMS	New Rural Cooperative Medical Scheme
NCDs	Non-Communicable Diseases
NHS	National Health Service
PIR	Poverty Income Ratio
PPS	Probability-Proportional-to-Size
PSU	Primary Sampling Unit
