# Supplementary material for: Inequities in the Hypertension and Diabetes Care Cascade: A Comparison of SES and Insurance in China, the US, and the UK
Source: Healthcare (Basel). 2026 Feb 15;14(4):501. doi: 10.3390/healthcare14040501 (PMC12940640; doi:10.3390/healthcare14040501)
Supplement: Supplementary file 1 [file healthcare-14-00501-s001.zip › healthcare-4093572-supplementary.pdf]

Supplementary Materials for:

Inequities in the Hypertension and Diabetes Care Cascade: A Comparison of SES and Insurance in China, the US, and the UK

Yutong Nie, Qiaorong Huang, Wentong Meng, Xue Li, Lei Chen, Xianming Mo*

Laboratory of Stem Cell Biology, Frontiers Science Center for Disease-related Molecular Network, Department of Neurology, West China Hospital, Sichuan University, Chengdu 610041, China.

***Correspondence:**

Xianming Mo

xmingmo@scu.edu.cn.

This supporting information includes:

Text S1. Detailed Survey Methodology.

S1.1. China (CHARLS)

S1.2. United States (NHANES)

S1.3. United Kingdom (ELSA)

Table S1. Variable harmonization strategy and definitions across China (CHARLS), the US (NHANES), and the UK (ELSA).

Table S2. Full multivariable logistic regression results for the association between socioeconomic status, covariates, and chronic disease management (Model 1 & Model 2).

Table S3. Sensitivity analysis of the associations between socioeconomic status and disease management using complete-case data (weighted and unweighted models).

Table S4. Sensitivity analysis of the associations between socioeconomic status and disease management restricted to participants aged ≥50 years.

Table S5. Sensitivity analysis of the associations between socioeconomic status and disease management adjusting for survey years/waves to control for secular trends.

Table S6. Derivation of analytic cohorts and participant exclusion flow due to missing physiological indicators.

Table S7. Step-by-step sample attrition and unweighted participant counts across the hypertension and diabetes care cascades.

Text S1. Detailed Survey Methodology

S1.1. China (CHARLS)

The China Health and Retirement Longitudinal Study (CHARLS) employed a multi-stage stratified probability-proportional-to-size (PPS) sampling method to ensure a nationally representative sample of residents aged 45 and older [16].

Sampling: The process involved four stages: county-level stratification by GDP and region; selection of administrative villages/communities (PSUs); listing and mapping of all households within PSUs; and random selection of households and individuals.

Biomarkers: Venous blood samples were collected by medically trained staff from the Chinese Center for Disease Control and Prevention (China CDC). HbA1c was measured using Boronate affinity HPLC, and plasma glucose was measured using the enzymatic colorimetric test.

Weighting: Individual weights accounting for household and individual non-response were applied. Specifically, biomarker weights were used to adjust for non-participation in the blood collection.

S1.2. United States (NHANES)

The National Health and Nutrition Examination Survey (NHANES) is a continuous cross-sectional survey utilizing a complex, four-stage probability sampling design to assess the health of the US civilian non-institutionalized population.

Cycles: We aggregated data from six continuous cycles (2007–2008 to 2017–2018). Detailed sampling designs for these periods have been described elsewhere [35–37].

Biomarkers: Data were collected via household interviews and standardized physical examinations in Mobile Examination Centers (MEC). Plasma glucose (Hexokinase method) and HbA1c (HPLC method) were measured at central laboratories.

Weighting: To account for the complex design and the aggregation of six survey cycles, we calculated a combined analytic weight by dividing the 2-year MEC weight (WTMEC2YR) by the number of cycles (N = 6), in accordance with NCHS analytical guidelines [37–39].

S1.3. United Kingdom (ELSA)

The English Longitudinal Study of Ageing (ELSA) is a panel study of the English population aged 50 and older, originally drawn from the Health Survey for England [17].

Waves: We pooled data from Waves 2, 4, 6, and 8, as these waves included nurse assessment visits for biomarker collection.

Biomarkers: Trained nurses collected venous blood samples during home visits. HbA1c was measured using HPLC methods standardized to IFCC guidelines.

Weighting: We applied the cross-sectional nurse weights (e.g., nurwt) to correct for non-response to the nurse visit and blood collection components.

Table S1. Variable harmonization strategy and definitions across China (CHARLS), US (NHANES), and UK (ELSA).

Domain / Variable	Harmonized Category (Analysis Level)	China (CHARLS)	US (NHANES)	UK (ELSA)
**Demographic characteristics**				
**Age**	Continuous (Years)			
**Gender**	Male / Female			
**Race / Ethnicity**		Han ethnicity and minorities.	Non-Hispanic White, Non-Hispanic Black, Mexican American, Other Hispanic, and Others.	White and Non-White.
**Health Behaviors**				
**BMI**	Continuous (kg/m²)			
**Smoking Status**	Ever Smoker: Yes.	"Have you ever chewed tobacco, smoked a pipe, smoked self-rolled cigarettes, or smoked cigarettes/cigars?"	"Have you smoked at least 100 cigarettes in your entire life?"	Self-reported history of ever smoking cigarettes.
	Never Smoker: No.			
**Alcohol Consumption**	Ever Drinker: Yes.	"Did you ever drink alcoholic beverages in the past?"	"In any one year, have you had at least 12 drinks of any type of alcoholic beverage?"	Self-reported history of ever drinking alcohol.
	Never Drinker: No.			
**Socioeconomic Status**				
**Education**	Low: Primary/Middle School or below	Low: Illiterate, primary, or middle school.	Low: Less than 9th grade or 9-11th grade (non-graduate).	Low: No qualifications or NVQ1.
	Medium: High School	Medium: High school or vocational school.	Medium: High school graduate or GED.	Medium: NVQ2/3, GCE O/A level, or equivalent.
	High: College or above	High: College, university, or post-graduate.	High: Some college or AA degree, college graduate or above.	High: Higher education, NVQ4/5, or degree.
**Economic Status**	Quartiles (Q1–Q4)	Indicator: Annual per capita household income.	Indicator: Poverty Income Ratio (PIR).	Indicator: Total non-pension household wealth.
	(Q1 = Poorest, Q4 = Wealthiest)	Method: Sum of all household income sources divided by household size.	Method: Ratio of family income to the federal poverty threshold (adjusted for family size).	Method: Sum of financial, housing, and physical wealth minus debts.
**Health Insurance**		Derived from *ins* & *ea001s1-s11*:	Derived from *HIQ031A-J* & *HIQ011*:	Derived from Econ Module:

**Insurance Classification**	1. No Insurance<br>(Uninsured)	(1) Uninsured: *ins*=0/2 (No insurance).	(1) Uninsured: *HIQ011*=No.	(1) NHS Only (Basic): Covered by National Health Service (Universal coverage, treated as base/public).
	2. Basic / Public<br>(China: Rural; US: Public; UK: NHS only)	(2) Rural (Low): *ea001s4*=1 (New Rural Cooperative Medical Scheme, NCMS).	(2) Public Insurance: Covered by Medicare, Medicaid, SCHIP, Military, Indian Health Service, State-sponsored, or other government plans (*HIQ031B, D-I*).	(2) Private: Covered by Private Medical Insurance.
	3. Intermediate / Mixed<br>(China: Resident/Other)	(3) Resident & Other (Mid): *ea001s2/3*=1 (Urban Resident) OR *ea001s6-11*=1 (Commercial/Other).	(3) Private Insurance: Covered by Private Insurance, Medi-Gap, or Single Service Plan (*HIQ031A, C, J*). (Priority Rule: If has Private -> Private; Else if has Public -> Public; Else -> Uninsured)	
	4. Advanced / Private<br>(China: Employee; US: Private; UK: Private)	(4) Employee (High): *ea001s1/5*=1 (Urban Employee or Public Medical Care).		

CHARLS = China Health and Retirement Longitudinal Study; NHANES = National Health and Nutrition Examination Survey; ELSA = English Longitudinal Study of Ageing; BMI = Body Mass Index; PIR = Poverty Income Ratio.

Table S2. Full multivariable logistic regression results for the association between socioeconomic status, covariates, and chronic disease management (Model 1 & Model 2).

Panel A. Hypertension Management

**Country/Variable**	Hypertension					
	Diagnosis		Treatment		Control	
	Model1	Model2	Model1	Model2	Model1	Model2
**China**						
**Education Level**						
Primary or below (Ref)	-	-	-	-	-	-
Middle/High School	0.98 (0.75, 1.29)	1.01 (0.77, 1.32)	1.06 (0.81, 1.39)	1.08 (0.82, 1.42)	1.02 (0.79, 1.32)	1 (0.77, 1.28)
College or above	1.58 (0.94, 2.66)	1.54 (0.91, 2.6)	1.92 (0.6, 6.18)	1.89 (0.57, 6.26)	0.93 (0.54, 1.6)	0.93 (0.53, 1.64)
**Household Income (Per Capita)**						
Quartile 1 (Lowest) (Ref)	-	-	-	-	-	-
Quartile 2	**1.23 (1.03, 1.46)**	**1.22 (1.01, 1.47)**	1.11 (0.95, 1.29)	1.12 (0.96, 1.3)	0.99 (0.84, 1.16)	0.99 (0.84, 1.16)
Quartile 3	0.98 (0.76, 1.26)	0.93 (0.69, 1.24)	1.17 (0.94, 1.46)	1.14 (0.92, 1.43)	0.96 (0.78, 1.18)	0.97 (0.79, 1.2)
Quartile 4 (Highest)	1.02 (0.82, 1.28)	0.96 (0.78, 1.19)	**1.56 (1.21, 2.02)**	**1.43 (1.11, 1.86)**	0.98 (0.76, 1.26)	1.05 (0.82, 1.33)
**Health Insurance Status**						
No Insurance (Ref)	-	-	-	-	-	-
Urban Employee Insurance	**2.79 (1.77, 4.39)**	**2.86 (1.61, 5.05)**	1.03 (0.66, 1.61)	0.98 (0.62, 1.55)	**1.4 (1, 1.97)**	1.41 (1, 1.99)
Urban Resident / Other	**2.79 (1.78, 4.36)**	**2.88 (1.66, 4.97)**	1.16 (0.83, 1.61)	1.1 (0.8, 1.52)	**1.49 (1.04,2.15)**	**1.57 (1.12, 2.19)**
New Rural Coop (NCMS)	**1.8 (1.2, 2.72)**	**1.96 (1.19, 3.25)**	0.98 (0.78, 1.24)	0.99 (0.79, 1.24)	1.28 (0.98, 1.66)	1.27 (0.97, 1.65)
**United States**						
**Education Level**						
< High School (Ref)	-	-	-	-	-	-
High School / GED	**0.81 (0.68, 0.98)**	**0.78 (0.65, 0.94)**	0.96 (0.75, 1.22)	0.92 (0.72, 1.18)	1.01 (0.85, 1.2)	1 (0.84, 1.2)
> High School	1.01 (0.84, 1.21)	0.97 (0.8, 1.17)	0.91 (0.7, 1.18)	0.84 (0.65, 1.1)	1.03 (0.87, 1.22)	1 (0.84, 1.18)
**Family Income (PIR)**						
Quartile 1 (Lowest) (Ref)	-	-	-	-	-	-
Quartile 2	1.05 (0.87, 1.26)	1.05 (0.87, 1.27)	0.94 (0.74, 1.2)	0.93 (0.73, 1.19)	0.96 (0.8, 1.14)	0.96 (0.81, 1.14)
Quartile 3	1.06 (0.89, 1.27)	1.08 (0.9, 1.28)	1.15 (0.88, 1.51)	1.15 (0.88, 1.51)	**1.32 (1.07, 1.62)**	**1.32 (1.07, 1.62)**

Quartile 4 (Highest)	0.86 (0.67, 1.1)	0.9 (0.71, 1.15)	1.2 (0.89, 1.6)	1.21 (0.9, 1.64)	**1.3 (1.03, 1.62)**	**1.3 (1.04, 1.62)**
**Health Insurance Status**						
Uninsured (Ref)	-	-	-	-	-	-
Public Insurance	**2 (1.56, 2.56)**	**1.99 (1.55, 2.57)**	**2.36 (1.82, 3.07)**	**2.34 (1.8, 3.05)**	**1.45 (1.1, 1.92)**	**1.47 (1.11, 1.94)**
Private Insurance	**1.46 (1.18, 1.82)**	**1.47 (1.17, 1.83)**	**2.63 (1.92, 3.59)**	**2.56 (1.87, 3.52)**	**1.44 (1.06, 1.96)**	**1.45 (1.06, 1.97)**
**United Kingdom**						
**Education Level**						
Primary or below (Ref)	-	-	-	-	-	-
Middle/High School	0.94 (0.86, 1.04)	0.97 (0.88, 1.08)	1.1 (0.99, 1.22)	1.05 (0.93, 1.17)	1.08 (0.96, 1.21)	1.1 (0.97, 1.23)
College or above	0.87 (0.76, 1)	0.95 (0.81, 1.1)	0.98 (0.84, 1.14)	0.96 (0.82, 1.13)	1.13 (0.95, 1.36)	1.16 (0.97, 1.38)
**Total Household Wealth**						
Quartile 1 (Lowest) (Ref)	-		-	-	-	-
Quartile 2	**0.88 (0.77, 1)**	**0.85 (0.73, 0.97)**	0.99 (0.87, 1.13)	**0.86 (0.74, 1)**	0.89 (0.76, 1.03)	0.9 (0.77, 1.05)
Quartile 3	0.91 (0.8, 1.03)	**0.83 (0.72, 0.96)**	1.1 (0.96, 1.27)	0.95 (0.81, 1.11)	0.91 (0.78, 1.06)	0.94 (0.8, 1.09)
Quartile 4 (Highest)	**0.8 (0.7, 0.91)**	**0.76 (0.66, 0.88)**	1.14 (0.99, 1.31)	0.98 (0.83, 1.14)	0.99 (0.84, 1.16)	1.02 (0.87, 1.2)
**Private Health Insurance**						
No (Public Only) (Ref)	-		-	-	-	-
Yes	1.02 (0.89, 1.17)	1.01 (0.87, 1.16)	**0.78 (0.68, 0.9)**	**0.83 (0.71, 0.96)**	1.01 (0.85, 1.19)	1.01 (0.86, 1.2)

Panel B. Diabetes Management

Country/Variable	Diabetes					
	Diagnosis		Treatment		Control	
	Model1	Model2	Model1	Model2	Model1	Model2
**China**						
**Education Level**						
Primary or below (Ref)	-	-	-	-	-	-
Middle/High School	0.77 (0.5, 1.19)	0.8 (0.51, 1.23)	0.79 (0.45, 1.39)	0.84 (0.49, 1.45)	1.67 (0.77, 3.63)	1.68 (0.78, 3.63)
College or above	0.93 (0.25, 3.38)	0.91 (0.26, 3.2)	1.03 (0.44, 2.4)	1 (0.43, 2.35)	1.42 (0.45, 4.47)	1.16 (0.4, 3.36)
**Household Income (Per Capita)**						
Quartile 1 (Lowest) (Ref)	-	-	-	-	-	-
Quartile 2	0.94 (0.65, 1.34)	0.94 (0.66, 1.32)	0.99 (0.7, 1.4)	1.03 (0.73, 1.46)	1.11 (0.7, 1.77)	1.13 (0.71, 1.79)
Quartile 3	1.12 (0.72, 1.74)	0.99 (0.67, 1.44)	1.41 (0.9, 2.19)	1.23 (0.82, 1.83)	**1.83 (1.08, 3.1)**	1.43 (0.91, 2.25)
Quartile 4 (Highest)	1.57 (1, 2.47)	1.51 (0.99, 2.31)	**1.95 (1.23, 3.09)**	**1.94 (1.21, 3.12)**	1.18 (0.7, 1.99)	1.14 (0.68, 1.91)
**Health Insurance Status**						
No Insurance (Ref)	-	-	-	-	-	-
Urban Employee Insurance	1.37 (0.78, 2.41)	1.33 (0.76, 2.34)	**0.49 (0.24, 0.98)**	0.51 (0.25, 1.04)	**0.3 (0.11, 0.8)**	**0.32 (0.12, 0.84)**
Urban Resident / Other	1.49 (0.77, 2.89)	1.24 (0.71, 2.17)	0.81 (0.35, 1.88)	0.64 (0.32, 1.29)	0.76 (0.23, 2.5)	0.5 (0.19, 1.37)
New Rural Coop (NCMS)	0.94 (0.61, 1.46)	0.91 (0.59, 1.39)	**0.46 (0.25, 0.83)**	**0.45 (0.25, 0.82)**	0.52 (0.22, 1.23)	0.53 (0.22, 1.24)
**United States**						
**Education Level**						
< High School (Ref)	-	-	-	-	-	-
High School / GED	0.98 (0.76, 1.26)	0.94 (0.71, 1.24)	0.94 (0.64, 1.37)	0.91 (0.6, 1.38)	0.8 (0.65, 1)	0.8 (0.63, 1.02)
> High School	0.96 (0.75, 1.22)	0.96 (0.72, 1.28)	0.98 (0.71, 1.36)	0.92 (0.67, 1.26)	0.93 (0.72, 1.2)	0.9 (0.69, 1.19)
**Family Income (PIR)**						
Quartile 1 (Lowest) (Ref)	-	-	-	-	-	-
Quartile 2	1.06 (0.83, 1.35)	1.11 (0.83, 1.48)	1.36 (0.99, 1.86)	1.3 (0.94, 1.8)	**1.3 (1.01, 1.66)**	**1.34 (1.04, 1.74)**
Quartile 3	1.03 (0.78, 1.36)	1.12 (0.84, 1.5)	1.16 (0.78, 1.71)	1.17 (0.74, 1.86)	1.21 (0.91, 1.6)	1.25 (0.92, 1.7)
Quartile 4 (Highest)	1.06 (0.76, 1.47)	1.17 (0.8, 1.7)	1.01 (0.65, 1.58)	1.19 (0.75, 1.89)	1.37 (0.98, 1.93)	**1.43 (1, 2.04)**

**Health Insurance Status**						
Uninsured (Ref)	-	-	-	-	-	-
Public Insurance	**1.81 (1.39, 2.36)**	**1.91 (1.39, 2.64)**	**1.96 (1.46, 2.65)**	**1.84 (1.32, 2.58)**	**1.44 (1.09, 1.9)**	**1.47 (1.09, 1.98)**
Private Insurance	**1.62 (1.24, 2.12)**	**1.56 (1.16, 2.1)**	**2.07 (1.38, 3.1)**	**1.85 (1.22, 2.81)**	1.3 (0.92, 1.86)	1.2 (0.82, 1.75)
**United Kingdom**						
**Education Level**						
Primary or below (Ref)	-	-	-	-	-	-
Middle/High School	1.21 (0.96, 1.53)	1.1 (0.85, 1.42)	**0.67 (0.49, 0.93)**	**0.66 (0.47, 0.95)**	1.06 (0.81, 1.4)	1.06 (0.81, 1.4)
College or above	**1.63 (1.08, 2.45)**	**1.76 (1.13, 2.75)**	0.75 (0.45, 1.23)	0.73 (0.43, 1.23)	0.77 (0.51, 1.16)	0.75 (0.5, 1.13)
**Total Household Wealth**						
Quartile 1 (Lowest) (Ref)	-	-	-	-	-	-
Quartile 2	1.27 (0.92, 1.74)	1.28 (0.89, 1.83)	1.34 (0.84, 2.15)	1.36 (0.8, 2.31)	1.38 (0.97, 1.97)	1.39 (0.97, 1.98)
Quartile 3	1.13 (0.81, 1.57)	1.08 (0.75, 1.57)	1.43 (0.9, 2.26)	1.36 (0.82, 2.27)	1.32 (0.92, 1.89)	1.33 (0.92, 1.93)
Quartile 4 (Highest)	1.11 (0.79, 1.55)	0.92 (0.63, 1.34)	1.03 (0.66, 1.61)	1.1 (0.66, 1.84)	**1.46 (1.02, 2.08)**	**1.46 (1.01, 2.12)**
**Private Health Insurance**						
No (Public Only) (Ref)	-	-	-	-	-	-
Yes	1.06 (0.73, 1.56)	1.14 (0.76, 1.72)	0.83 (0.49, 1.41)	1.04 (0.56, 1.92)	1.31 (0.86, 2)	1.32 (0.86, 2.02)

Data Presentation: Values are Odds Ratios (OR) with 95% Confidence Intervals (CI). Models: Model 1 is adjusted for age, gender, and race. Model 2 is further adjusted for smoking status, alcohol consumption, and BMI. Significance: Bold values indicate statistical significance ($p < 0.05$).

Table S3. Sensitivity analysis of the associations between socioeconomic status and disease management using complete-case data (weighted and unweighted models).

Panel A. Hypertension Management

Country/Variable	Hypertension					
	Diagnosis		Treatment		Control	
	Weighted	Unweighted	Weighted	Unweighted	Weighted	Unweighted
**China**						
**Education Level**						
Primary or below (Ref)	-	-	-	-	-	-
Middle/High School	0.98 (0.73, 1.33)	0.97 (0.79, 1.19)	1.06 (0.77, 1.46)	1.02 (0.8, 1.31)	0.9 (0.64, 1.28)	0.83 (0.64, 1.07)
College or above	1.62 (0.85, 3.09)	1.14 (0.67, 1.95)	1.3 (0.6, 2.82)	1.1 (0.57, 2.12)	1.59 (0.79, 3.23)	1.29 (0.73, 2.28)
**Household Income (Per Capita)**						
Quartile 1 (Lowest) (Ref)	-	-	-	-	-	-
Quartile 2	**1.17 (1.01, 1.36)**	**1.19 (1.03, 1.36)**	1.01 (0.84, 1.2)	0.95 (0.8, 1.12)	1.13 (0.93, 1.38)	0.91 (0.76, 1.09)
Quartile 3	0.98 (0.81, 1.19)	0.98 (0.86, 1.13)	1.09 (0.88, 1.36)	1.02 (0.86, 1.21)	1.15 (0.85, 1.57)	**1.25 (1.04, 1.49)**
Quartile 4 (Highest)	1.07 (0.86, 1.34)	0.94 (0.81, 1.09)	**1.3 (1, 1.69)**	1.04 (0.87, 1.25)	1.07 (0.78, 1.47)	1.15 (0.95, 1.4)
**Health Insurance Status**						
No Insurance (Ref)	-	-	-	-	-	-
Urban Employee Insurance	**1.66 (1.07, 2.58)**	**1.59 (1.22, 2.08)**	1.45 (0.95, 2.22)	**1.6 (1.14, 2.23)**	1.52 (0.92, 2.51)	1.09 (0.77, 1.55)
Urban Resident / Other	1.52 (0.97, 2.37)	**1.41 (1.08, 1.84)**	1.47 (0.96, 2.26)	1.25 (0.9, 1.74)	1.5 (0.88, 2.56)	1.18 (0.83, 1.7)
New Rural Coop (NCMS)	1.28 (0.89, 1.82)	1.18 (0.96, 1.46)	1.23 (0.87, 1.74)	1.19 (0.91, 1.56)	1.16 (0.74, 1.82)	0.96 (0.71, 1.3)
**United States**						
**Education Level**						
< High School (Ref)	-	-	-	-	-	-
High School / GED	0.85 (0.7, 1.05)	1 (0.84, 1.19)	0.96 (0.7, 1.31)	0.84 (0.66, 1.06)	0.93 (0.75, 1.15)	1.04 (0.88, 1.21)
> High School	1.04 (0.81, 1.33)	1.16 (0.98, 1.37)	0.79 (0.57, 1.08)	**0.78 (0.63, 0.97)**	0.99 (0.81, 1.2)	1.05 (0.9, 1.22)
**Family Income (PIR)**						
Quartile 1 (Lowest) (Ref)	-	-	-	-	-	-
Quartile 2	0.95 (0.77, 1.17)	0.92 (0.77, 1.1)	1 (0.73, 1.36)	1.02 (0.82, 1.28)	0.97 (0.77, 1.22)	1.02 (0.87, 1.2)
Quartile 3	0.99 (0.79, 1.23)	0.87 (0.72, 1.05)	1.14 (0.83, 1.56)	1.09 (0.85, 1.39)	1.13 (0.89, 1.44)	1.08 (0.91, 1.29)

Quartile 4 (Highest)	0.78 (0.59, 1.04)	0.91 (0.74, 1.11)	1.29 (0.9, 1.85)	1.22 (0.93, 1.59)	1.16 (0.86, 1.57)	**1.23 (1.01, 1.48)**
**Health Insurance Status**						
Uninsured (Ref)	-	-	-	-	-	-
Public Insurance	**2.26 (1.63, 3.13)**	**2.14 (1.77, 2.59)**	**2.4 (1.72, 3.35)**	**2.11 (1.68, 2.66)**	**1.48 (1.11, 1.97)**	**1.3 (1.06, 1.61)**
Private Insurance	**1.66 (1.25, 2.2)**	**1.55 (1.29, 1.86)**	**2.74 (1.9, 3.94)**	**2.37 (1.88, 2.99)**	**1.49 (1.05, 2.1)**	**1.49 (1.2, 1.84)**
**United Kingdom**						
**Education Level**						
Primary or below (Ref)	-	-	-	-	-	-
Middle/High School	1 (0.9, 1.11)	0.99 (0.9, 1.09)	1.09 (0.97, 1.22)	**1.12 (1, 1.25)**	1.29 (0.37, 4.53)	1.17 (0.24, 5.73)
College or above	0.99 (0.85, 1.16)	1.03 (0.89, 1.18)	1.02 (0.86, 1.2)	1.1 (0.94, 1.29)	2.27 (0.43, 12.06)	2.08 (0.27, 16.1)
**Total Household Wealth**						
Quartile 1 (Lowest) (Ref)	-	-	-	-	-	-
Quartile 2	0.92 (0.8, 1.06)	**0.85 (0.75, 0.95)**	0.97 (0.83, 1.13)	1.03 (0.91, 1.17)	1.75 (0.22, 13.89)	0.55 (0.05, 6.22)
Quartile 3	0.93 (0.8, 1.07)	**0.87 (0.77, 0.99)**	1.11 (0.94, 1.3)	1.06 (0.93, 1.22)	2.49 (0.42, 14.94)	2.39 (0.4, 14.42)
Quartile 4 (Highest)	0.87 (0.75, 1.01)	**0.87 (0.76, 0.99)**	**1.19 (1.01, 1.4)**	**1.25 (1.08, 1.44)**	1.08 (0.14, 8.31)	1.22 (0.14, 10.86)
**Private Health Insurance**						
No (Public Only) (Ref)	-	-	-	-	-	-
Yes	1 (0.87, 1.16)	1 (0.88, 1.14)	**0.81 (0.69, 0.95)**	**0.83 (0.72, 0.96)**	NC	NC

Panel B. Diabetes Management

Country/Variable	Diabetes					
	Diagnosis		Treatment		Control	
	Weighted	Unweighted	Weighted	Unweighted	Weighted	Unweighted
**China**						
**Education Level**						
Primary or below (Ref)	-	-	-	-	-	-
Middle/High School	**1.97 (1.18, 3.28)**	1.46 (0.95, 2.23)	0.93 (0.44, 1.94)	0.86 (0.49, 1.52)	2.33 (0.81, 6.69)	1.11 (0.55, 2.23)
College or above	**4.07 (1.08, 15.34)**	2.97 (0.96, 9.21)	0.71 (0.25, 2.03)	0.95 (0.32, 2.82)	0.76 (0.16, 3.48)	0.42 (0.11, 1.56)
**Household Income (Per Capita)**						
Quartile 1 (Lowest) (Ref)	-	-	-	-	-	-
Quartile 2	1.03 (0.75, 1.41)	1.07 (0.8, 1.44)	1.14 (0.73, 1.78)	1.03 (0.67, 1.59)	1 (0.54, 1.84)	0.98 (0.54, 1.77)
Quartile 3	1.16 (0.78, 1.71)	0.93 (0.69, 1.26)	1.54 (0.89, 2.67)	1.55 (0.99, 2.42)	1.65 (0.85, 3.18)	1.33 (0.75, 2.33)
Quartile 4 (Highest)	1.43 (0.89, 2.3)	1.14 (0.83, 1.57)	**3.76 (1.86, 7.58)**	**1.61 (1.01, 2.58)**	1.41 (0.64, 3.1)	1.4 (0.78, 2.51)
**Health Insurance Status**						
No Insurance (Ref)	-	-	-	-	-	-
Urban Employee Insurance	1.46 (0.59, 3.58)	**2.17 (1.24, 3.8)**	0.38 (0.12, 1.2)	0.62 (0.24, 1.62)	0.31 (0.07, 1.36)	1.71 (0.67, 4.36)
Urban Resident / Other	1.38 (0.62, 3.07)	1.72 (0.99, 2.98)	**0.28 (0.09, 0.83)**	0.45 (0.17, 1.18)	0.26 (0.06, 1.22)	1.37 (0.52, 3.61)
New Rural Coop (NCMS)	0.87 (0.44, 1.73)	1.22 (0.77, 1.94)	**0.23 (0.09, 0.59)**	**0.39 (0.16, 0.93)**	0.5 (0.15, 1.68)	1.45 (0.63, 3.34)
**United States**						
**Education Level**						
< High School (Ref)	-	-	-	-	-	-
High School / GED	0.88 (0.65, 1.17)	0.84 (0.68, 1.05)	0.89 (0.55, 1.45)	0.92 (0.69, 1.24)	0.79 (0.59, 1.05)	1.02 (0.82, 1.28)
> High School	0.95 (0.71, 1.27)	1.03 (0.84, 1.28)	0.96 (0.68, 1.36)	0.93 (0.71, 1.23)	0.92 (0.67, 1.25)	1.08 (0.88, 1.33)
**Family Income (PIR)**						
Quartile 1 (Lowest) (Ref)	-	-	-	-	-	-
Quartile 2	1.07 (0.79, 1.44)	0.93 (0.74, 1.17)	1.25 (0.89, 1.76)	0.95 (0.71, 1.27)	**1.34 (1.01, 1.77)**	**1.32 (1.05, 1.65)**
Quartile 3	1.18 (0.88, 1.6)	1.02 (0.8, 1.29)	1.08 (0.66, 1.79)	0.96 (0.71, 1.31)	1.33 (0.93, 1.9)	1.14 (0.9, 1.45)
Quartile 4 (Highest)	1.1 (0.74, 1.63)	1.06 (0.81, 1.38)	1.14 (0.69, 1.87)	1.06 (0.75, 1.5)	1.32 (0.88, 1.98)	**1.34 (1.03, 1.74)**

**Health Insurance Status**						
Uninsured (Ref)	-	-	-	-	-	-
Public Insurance	**1.83 (1.31, 2.55)**	**1.92 (1.51, 2.46)**	**1.97 (1.35, 2.86)**	**1.98 (1.46, 2.67)**	**1.48 (1.08, 2.02)**	1.29 (0.98, 1.71)
Private Insurance	**1.53 (1.13, 2.07)**	**1.47 (1.15, 1.87)**	**1.99 (1.24, 3.2)**	**2.01 (1.47, 2.75)**	1.06 (0.69, 1.62)	1.02 (0.76, 1.35)
**United Kingdom**						
**Education Level**						
Primary or below (Ref)	-	-	-	-	-	-
Middle/High School	1.15 (0.89, 1.49)	1.17 (0.92, 1.49)	0.7 (0.49, 1)	0.84 (0.59, 1.2)	1.03 (0.76, 1.39)	0.99 (0.75, 1.3)
College or above	**1.89 (1.19, 3.03)**	**1.74 (1.18, 2.58)**	0.74 (0.43, 1.26)	0.87 (0.52, 1.45)	0.72 (0.45, 1.16)	0.88 (0.58, 1.34)
**Total Household Wealth**						
Quartile 1 (Lowest) (Ref)	-	-	-	-	-	-
Quartile 2	1.38 (0.95, 1.99)	**1.38 (1.02, 1.87)**	1.59 (0.93, 2.71)	1.36 (0.87, 2.11)	1.32 (0.87, 1.99)	1.16 (0.83, 1.63)
Quartile 3	1.22 (0.84, 1.77)	1.24 (0.91, 1.69)	1.65 (0.99, 2.76)	1.3 (0.83, 2.04)	1.32 (0.87, 2)	1.13 (0.79, 1.6)
Quartile 4 (Highest)	1.13 (0.77, 1.65)	0.95 (0.69, 1.3)	1.53 (0.9, 2.57)	1.06 (0.67, 1.68)	1.39 (0.91, 2.12)	1.31 (0.89, 1.91)
**Private Health Insurance**						
No (Public Only) (Ref)	-	-	-	-	-	-
Yes	1.02 (0.67, 1.57)	1 (0.68, 1.47)	0.88 (0.46, 1.66)	0.97 (0.57, 1.66)	1.08 (0.67, 1.75)	1.11 (0.71, 1.74)

Data are presented as Odds Ratios (OR) with 95% Confidence Intervals (CI). Bold values indicate statistical significance ($p < 0.05$). Models: Results are based on complete-case analysis (no imputation). The "Weighted" columns apply sampling weights; the "Unweighted" columns do not. All models (Model 2) are adjusted for age, sex, race, smoking status, alcohol consumption, and BMI. Abbreviations: NC, not calculated. Specific Note: Estimates for private health insurance in the UK regarding hypertension control were not calculated (NC) due to insufficient sample size and model non-convergence.

Table S4. Sensitivity analysis of the associations between socioeconomic status and disease management restricted to participants aged ≥50 years.

Panel A. Hypertension Management

Country/Variable	Hypertension					
	Diagnosis		Treatment		Control	
	Main Model	Age ≥50	Main Model	Age ≥50	Main Model	Age ≥50
**China**						
**Education Level**						
Primary or below (Ref)	-	-	-	-	-	-
Middle/High School	1.01 (0.77, 1.32)	1.01 (0.75, 1.36)	1.08 (0.82, 1.42)	1.1 (0.82, 1.49)	1 (0.77, 1.28)	0.91 (0.71, 1.17)
College or above	1.54 (0.91, 2.6)	**1.78 (1, 3.17)**	1.89 (0.57, 6.26)	2.02 (0.56, 7.34)	0.93 (0.53, 1.64)	0.84 (0.46, 1.55)
**Household Income (Per Capita)**						
Quartile 1 (Lowest) (Ref)	-	-	-	-	-	-
Quartile 2	**1.22 (1.01, 1.47)**	1.2 (0.99, 1.46)	1.12 (0.96, 1.3)	1.11 (0.95, 1.3)	0.99 (0.84, 1.16)	1.01 (0.85, 1.19)
Quartile 3	0.93 (0.69, 1.24)	0.94 (0.69, 1.28)	1.14 (0.92, 1.43)	1.13 (0.89, 1.42)	0.97 (0.79, 1.2)	0.98 (0.78, 1.21)
Quartile 4 (Highest)	0.96 (0.78, 1.19)	1.03 (0.82, 1.29)	**1.43 (1.11, 1.86)**	**1.45 (1.09, 1.93)**	1.05 (0.82, 1.33)	1.03 (0.8, 1.33)
**Health Insurance Status**						
No Insurance (Ref)	-	-	-	-	-	-
Urban Employee Insurance	**2.86 (1.61, 5.05)**	**2.3 (1.22, 4.34)**	0.98 (0.62, 1.55)	0.89 (0.55, 1.44)	1.41 (1, 1.99)	1.54 (1.11, 2.14)
Urban Resident / Other	**2.88 (1.66, 4.97)**	**2.56 (1.41, 4.62)**	1.1 (0.8, 1.52)	1.11 (0.79, 1.56)	**1.57 (1.12, 2.19)**	**1.54 (1.08, 2.19)**
New Rural Coop (NCMS)	**1.96 (1.19, 3.25)**	1.71 (0.99, 2.97)	0.99 (0.79, 1.24)	1.01 (0.8, 1.28)	1.27 (0.97, 1.65)	1.22 (0.92, 1.61)
**United States**						
**Education Level**						
< High School (Ref)	-	-	-	-	-	-
High School / GED	**0.78 (0.65, 0.94)**	0.79 (0.62, 1)	0.92 (0.72, 1.18)	0.85 (0.61, 1.18)	1 (0.84, 1.2)	0.94 (0.78, 1.12)
> High School	0.97 (0.8, 1.17)	0.89 (0.71, 1.12)	0.84 (0.65, 1.1)	0.73 (0.51, 1.04)	1 (0.84, 1.18)	0.98 (0.83, 1.17)
**Family Income (PIR)**						
Quartile 1 (Lowest) (Ref)	-	-	-	-	-	-
Quartile 2	1.05 (0.87, 1.27)	1.02 (0.8, 1.3)	0.93 (0.73, 1.19)	0.89 (0.66, 1.21)	0.96 (0.81, 1.14)	1.04 (0.86, 1.26)
Quartile 3	1.08 (0.9, 1.28)	1.04 (0.82, 1.32)	1.15 (0.88, 1.51)	1.04 (0.76, 1.42)	**1.32 (1.07, 1.62)**	**1.37 (1.08, 1.73)**

Quartile 4 (Highest)	0.9 (0.71, 1.15)	0.96 (0.68, 1.36)	1.21 (0.9, 1.64)	1.07 (0.75, 1.54)	**1.3 (1.04, 1.62)**	**1.34 (1.02, 1.75)**
**Health Insurance Status**						
Uninsured (Ref)	-	-	-	-	-	-
Public Insurance	**1.99 (1.55, 2.57)**	**1.94 (1.41, 2.68)**	**2.34 (1.8, 3.05)**	**2.61 (1.84, 3.72)**	**1.47 (1.11, 1.94)**	**1.41 (1.02, 1.96)**
Private Insurance	**1.47 (1.17, 1.83)**	**1.41 (1.05, 1.89)**	**2.56 (1.87, 3.52)**	**3.29 (2.15, 5.05)**	**1.45 (1.06, 1.97)**	1.38 (0.97, 1.95)
**United Kingdom**						
**Education Level**						
Primary or below (Ref)	-	-	-	-	-	-
Middle/High School	0.97 (0.88, 1.08)	0.96 (0.86, 1.06)	1.05 (0.93, 1.17)	1.09 (0.99, 1.21)	1.1 (0.97, 1.23)	0.96 (0.34, 2.7)
College or above	0.95 (0.81, 1.1)	0.95 (0.82, 1.1)	0.96 (0.82, 1.13)	0.96 (0.82, 1.11)	1.16 (0.97, 1.38)	1.26 (0.25, 6.36)
**Total Household Wealth**						
Quartile 1 (Lowest) (Ref)		-	-	-	-	-
Quartile 2	**0.85 (0.73, 0.97)**	**0.82 (0.72, 0.94)**	**0.86 (0.74, 1)**	1 (0.88, 1.13)	0.9 (0.77, 1.05)	1.41 (0.27, 7.24)
Quartile 3	**0.83 (0.72, 0.96)**	**0.8 (0.7, 0.93)**	0.95 (0.81, 1.11)	1.11 (0.97, 1.27)	0.94 (0.8, 1.09)	2.09 (0.5, 8.7)
Quartile 4 (Highest)	**0.76 (0.66, 0.88)**	**0.71 (0.62, 0.82)**	0.98 (0.83, 1.14)	**1.25 (1.09, 1.44)**	1.02 (0.87, 1.2)	1.2 (0.24, 5.95)
**Private Health Insurance**						
No (Public Only) (Ref)		-	-	-	-	-
Yes	1.01 (0.87, 1.16)	1.04 (0.9, 1.2)	**0.83 (0.71, 0.96)**	**0.76 (0.66, 0.87)**	1.01 (0.86, 1.2)	1 (0.11, 9.27)

Panel B. Diabetes Management

Country/Variable	Diabetes					
	Diagnosis		Treatment		Control	
	Main Model	Age ≥50	Main Model	Age ≥50	Main Model	Age ≥50
**China**						
**Education Level**						
Primary or below (Ref)	-	-	-	-	-	-
Middle/High School	0.8 (0.51, 1.23)	0.75 (0.47, 1.2)	0.84 (0.49, 1.45)	0.9 (0.5, 1.61)	1.67 (0.77, 3.63)	1.76 (0.78, 3.98)
College or above	0.91 (0.26, 3.2)	0.94 (0.22, 3.97)	1 (0.43, 2.35)	1.08 (0.43, 2.67)	1.42 (0.45, 4.47)	1.19 (0.39, 3.66)
**Household Income (Per Capita)**						
Quartile 1 (Lowest) (Ref)	-	-	-	-	-	-
Quartile 2	0.94 (0.66, 1.32)	0.91 (0.63, 1.31)	1.03 (0.73, 1.46)	1.06 (0.74, 1.51)	1.11 (0.7, 1.77)	1.18 (0.73, 1.91)
Quartile 3	0.99 (0.67, 1.44)	1.02 (0.68, 1.53)	1.23 (0.82, 1.83)	1.24 (0.82, 1.88)	**1.83 (1.08, 3.1)**	1.49 (0.93, 2.37)
Quartile 4 (Highest)	1.51 (0.99, 2.31)	1.57 (1, 2.49)	**1.94 (1.21, 3.12)**	**2.01 (1.22, 3.31)**	1.18 (0.7, 1.99)	1.17 (0.68, 2.02)
**Health Insurance Status**						
No Insurance (Ref)	-	-	-	-	-	-
Urban Employee Insurance	1.33 (0.76, 2.34)	1.31 (0.73, 2.36)	0.51 (0.25, 1.04)	0.5 (0.24, 1.05)	**0.3 (0.11, 0.8)**	**0.31 (0.12, 0.85)**
Urban Resident / Other	1.24 (0.71, 2.17)	1.29 (0.71, 2.33)	0.64 (0.32, 1.29)	0.59 (0.28, 1.25)	0.76 (0.23, 2.5)	0.51 (0.18, 1.48)
New Rural Coop (NCMS)	0.91 (0.59, 1.39)	0.93 (0.59, 1.46)	**0.45 (0.25, 0.82)**	**0.44 (0.23, 0.82)**	0.52 (0.22, 1.23)	0.54 (0.22, 1.34)
**United States**						
**Education Level**						
< High School (Ref)	-	-	-	-	-	-
High School / GED	0.94 (0.71, 1.24)	0.96 (0.68, 1.37)	0.91 (0.6, 1.38)	1 (0.6, 1.66)	0.8 (0.65, 1)	0.79 (0.56, 1.1)
> High School	0.96 (0.72, 1.28)	0.94 (0.68, 1.31)	0.92 (0.67, 1.26)	0.91 (0.61, 1.34)	0.93 (0.72, 1.2)	1.12 (0.79, 1.59)
**Family Income (PIR)**						
Quartile 1 (Lowest) (Ref)	-	-	-	-	-	-
Quartile 2	1.11 (0.83, 1.48)	1.19 (0.82, 1.73)	1.3 (0.94, 1.8)	1.13 (0.79, 1.63)	**1.3 (1.01, 1.66)**	1.04 (0.77, 1.42)
Quartile 3	1.12 (0.84, 1.5)	1.18 (0.79, 1.77)	1.17 (0.74, 1.86)	0.96 (0.54, 1.72)	1.21 (0.91, 1.6)	0.86 (0.59, 1.25)
Quartile 4 (Highest)	1.17 (0.8, 1.7)	1.08 (0.71, 1.66)	1.19 (0.75, 1.89)	1.06 (0.58, 1.94)	1.37 (0.98, 1.93)	1.21 (0.73, 2)

**Health Insurance Status**						
Uninsured (Ref)	-	-	-	-	-	-
Public Insurance	**1.91 (1.39, 2.64)**	**1.72 (1.11, 2.66)**	**1.84 (1.32, 2.58)**	1.4 (0.77, 2.55)	**1.44 (1.09, 1.9)**	1.18 (0.73, 1.9)
Private Insurance	**1.56 (1.16, 2.1)**	1.54 (0.99, 2.42)	**1.85 (1.22, 2.81)**	1.35 (0.66, 2.79)	1.3 (0.92, 1.86)	1.04 (0.62, 1.76)
**United Kingdom**						
**Education Level**						
Primary or below (Ref)	-	-	-	-	-	-
Middle/High School	1.1 (0.85, 1.42)	1.11 (0.86, 1.43)	**0.66 (0.47, 0.95)**	**0.69 (0.49, 0.97)**	1.06 (0.81, 1.4)	1.06 (0.79, 1.42)
College or above	**1.76 (1.13, 2.75)**	**1.81 (1.14, 2.87)**	0.73 (0.43, 1.23)	0.84 (0.5, 1.4)	0.77 (0.51, 1.16)	0.73 (0.46, 1.15)
**Total Household Wealth**						
Quartile 1 (Lowest) (Ref)	-	-	-	-	-	-
Quartile 2	1.28 (0.89, 1.83)	1.41 (0.98, 2.03)	1.36 (0.8, 2.31)	1.56 (0.94, 2.57)	1.38 (0.97, 1.97)	1.4 (0.93, 2.1)
Quartile 3	1.08 (0.75, 1.57)	1.24 (0.86, 1.79)	1.36 (0.82, 2.27)	1.58 (0.97, 2.56)	1.32 (0.92, 1.89)	1.34 (0.89, 2.01)
Quartile 4 (Highest)	0.92 (0.63, 1.34)	1.12 (0.77, 1.64)	1.1 (0.66, 1.84)	1.44 (0.88, 2.35)	**1.46 (1.02, 2.08)**	1.44 (0.95, 2.19)
**Private Health Insurance**						
No (Public Only) (Ref)	-	-	-	-	-	-
Yes	1.14 (0.76, 1.72)	1.08 (0.71, 1.64)	1.04 (0.56, 1.92)	0.87 (0.49, 1.55)	1.31 (0.86, 2)	1.16 (0.72, 1.87)

Note: Data presentation: Data are presented as adjusted Odds Ratios (aOR) with 95% Confidence Intervals (CI). Boldface indicates statistical significance at $P < 0.05$. Models: Estimates were derived from survey-weighted multivariable logistic regression models. Adjustments: All models were adjusted for age, gender, race/ethnicity (US/China), smoking status, alcohol consumption, and BMI. Missing Data: Missing data for covariates were imputed using Multiple Imputation by Chained Equations (MICE). However, participants with missing data for the specific outcome variable (Diagnosis, Treatment, or Control) were excluded from the analysis (listwise deletion) to ensure the robustness of the estimates. Rationale: The sensitivity analysis restricted to participants aged ≥50 years was conducted to assess whether the observed disparities persist in the population with the highest burden of chronic disease and to minimize the potential confounding effect of early-onset disease types.

Table S5. Sensitivity analysis of the associations between socioeconomic status and disease management adjusting for survey years/waves to control for secular trends.

Panel A. Hypertension Management

Country/Variable	Hypertension					
	Diagnosis		Treatment		Control	
	Main Model	+Year Adj.	Main Model	+Year Adj.	Main Model	+Year Adj.
**China**						
**Education Level**						
Primary or below (Ref)	-	-	-	-	-	-
Middle/High School	1.01 (0.77, 1.32)	0.99 (0.75, 1.3)	1.08 (0.82, 1.42)	1.07 (0.82, 1.41)	1 (0.77, 1.28)	1 (0.77, 1.29)
College or above	1.54 (0.91, 2.6)	1.52 (0.89, 2.6)	1.89 (0.57, 6.26)	1.77 (0.59, 5.28)	0.93 (0.53, 1.64)	0.93 (0.52, 1.66)
**Household Income (Per Capita)**						
Quartile 1 (Lowest) (Ref)	-	-	-	-	-	-
Quartile 2	**1.22 (1.01, 1.47)**	**1.26 (1.06, 1.5)**	1.12 (0.96, 1.3)	1.07 (0.93, 1.24)	0.99 (0.84, 1.16)	**1.17 (1.01, 1.35)**
Quartile 3	0.93 (0.69, 1.24)	1.08 (0.82, 1.43)	1.14 (0.92, 1.43)	1.18 (0.94, 1.48)	0.97 (0.79, 1.2)	1.06 (0.87, 1.3)
Quartile 4 (Highest)	0.96 (0.78, 1.19)	1.12 (0.91, 1.37)	**1.43 (1.11, 1.86)**	**1.34 (1.05, 1.7)**	1.05 (0.82, 1.33)	1.1 (0.87, 1.4)
**Health Insurance Status**						
No Insurance (Ref)	-	-	-	-	-	-
Urban Employee Insurance	**2.86 (1.61, 5.05)**	**2.86 (1.62, 5.06)**	0.98 (0.62, 1.55)	1 (0.64, 1.57)	1.41 (1, 1.99)	**1.45 (1.03, 2.05)**
Urban Resident / Other	**2.88 (1.66, 4.97)**	**2.68 (1.52, 4.71)**	1.1 (0.8, 1.52)	1.13 (0.82, 1.55)	**1.57 (1.12, 2.19)**	**1.61 (1.15, 2.25)**
New Rural Coop (NCMS)	**1.96 (1.19, 3.25)**	**2.12 (1.29, 3.49)**	0.99 (0.79, 1.24)	1.03 (0.82, 1.3)	1.27 (0.97, 1.65)	1.3 (1, 1.69)
**United States**						
**Education Level**						
< High School (Ref)	-	-	-	-	-	-
High School / GED	**0.78 (0.65, 0.94)**	**0.81 (0.66, 0.99)**	0.92 (0.72, 1.18)	0.95 (0.74, 1.22)	1 (0.84, 1.2)	1.03 (0.87, 1.23)
> High School	0.97 (0.8, 1.17)	0.97 (0.77, 1.23)	0.84 (0.65, 1.1)	0.86 (0.66, 1.13)	1 (0.84, 1.18)	1.04 (0.87, 1.23)
**Family Income (PIR)**						
Quartile 1 (Lowest) (Ref)	-	-	-	-	-	-
Quartile 2	1.05 (0.87, 1.27)	0.99 (0.8, 1.22)	0.93 (0.73, 1.19)	0.94 (0.73, 1.19)	0.96 (0.81, 1.14)	0.92 (0.76, 1.11)
Quartile 3	1.08 (0.9, 1.28)	0.98 (0.8, 1.22)	1.15 (0.88, 1.51)	1.1 (0.84, 1.46)	**1.32 (1.07, 1.62)**	1.18 (0.94, 1.48)

Quartile 4 (Highest)	0.9 (0.71, 1.15)	0.78 (0.6, 1.02)	1.21 (0.9, 1.64)	1.28 (0.93, 1.74)	**1.3 (1.04, 1.62)**	1.19 (0.95, 1.5)
**Health Insurance Status**						
Uninsured (Ref)	-	-	-	-	-	-
Public Insurance	**1.99 (1.55, 2.57)**	**2.32 (1.68, 3.21)**	**2.34 (1.8, 3.05)**	**2.55 (1.95, 3.34)**	**1.47 (1.11, 1.94)**	**1.49 (1.12, 1.97)**
Private Insurance	**1.47 (1.17, 1.83)**	**1.64 (1.25, 2.16)**	**2.56 (1.87, 3.52)**	**2.62 (1.92, 3.58)**	**1.45 (1.06, 1.97)**	**1.51 (1.11, 2.06)**
**United Kingdom**						
**Education Level**						
Primary or below (Ref)	-	-	-	-	-	-
Middle/High School	0.97 (0.88, 1.08)	0.93 (0.84, 1.03)	1.05 (0.93, 1.17)	0.93 (0.83, 1.04)	1.1 (0.97, 1.23)	0.95 (0.33, 2.75)
College or above	0.95 (0.81, 1.1)	0.93 (0.8, 1.08)	0.96 (0.82, 1.13)	**0.81 (0.69, 0.95)**	1.16 (0.97, 1.38)	1.21 (0.24, 6.07)
**Total Household Wealth**						
Quartile 1 (Lowest) (Ref)		-	-	-	-	-
Quartile 2	**0.85 (0.73, 0.97)**	**0.83 (0.72, 0.95)**	**0.86 (0.74, 1)**	1.08 (0.94, 1.24)	0.9 (0.77, 1.05)	1.43 (0.28, 7.36)
Quartile 3	**0.83 (0.72, 0.96)**	**0.8 (0.7, 0.92)**	0.95 (0.81, 1.11)	1.09 (0.95, 1.26)	0.94 (0.8, 1.09)	2.13 (0.52, 8.72)
Quartile 4 (Highest)	**0.76 (0.66, 0.88)**	**0.69 (0.6, 0.8)**	0.98 (0.83, 1.14)	1.07 (0.92, 1.25)	1.02 (0.87, 1.2)	1.21 (0.24, 6.1)
**Private Health Insurance**						
No (Public Only) (Ref)		-	-	-	-	-
Yes	1.01 (0.87, 1.16)	1.06 (0.92, 1.23)	**0.83 (0.71, 0.96)**	0.87 (0.74, 1.01)	1.01 (0.86, 1.2)	0.97 (0.11, 8.89)

Panel B. Diabetes Management

Country/Variable	Diabetes					
	Diagnosis		Treatment		Control	
	Main Model	+Year Adj.	Main Model	+Year Adj.	Main Model	+Year Adj.
**China**						
**Education Level**						
Primary or below (Ref)	-	-	-	-	-	-
Middle/High School	0.8 (0.51, 1.23)	0.77 (0.48, 1.25)	0.84 (0.49, 1.45)	0.87 (0.49, 1.55)	1.67 (0.77, 3.63)	1.68 (0.83, 3.38)
College or above	0.91 (0.26, 3.2)	0.81 (0.19, 3.5)	1 (0.43, 2.35)	1.04 (0.45, 2.43)	1.42 (0.45, 4.47)	1.34 (0.46, 3.94)
**Household Income (Per Capita)**						
Quartile 1 (Lowest) (Ref)	-	-	-	-	-	-
Quartile 2	0.94 (0.66, 1.32)	1.13 (0.91, 1.41)	1.03 (0.73, 1.46)	0.9 (0.65, 1.25)	1.11 (0.7, 1.77)	0.98 (0.64, 1.49)
Quartile 3	0.99 (0.67, 1.44)	1.06 (0.82, 1.38)	1.23 (0.82, 1.83)	**1.63 (1.08, 2.46)**	**1.83 (1.08, 3.1)**	1.39 (0.84, 2.3)
Quartile 4 (Highest)	1.51 (0.99, 2.31)	1.26 (0.88, 1.79)	**1.94 (1.21, 3.12)**	**1.76 (1.07, 2.91)**	1.18 (0.7, 1.99)	1.04 (0.59, 1.81)
**Health Insurance Status**						
No Insurance (Ref)	-	-	-	-	-	-
Urban Employee Insurance	1.33 (0.76, 2.34)	1.42 (0.77, 2.64)	0.51 (0.25, 1.04)	**0.46 (0.22, 0.96)**	**0.3 (0.11, 0.8)**	**0.36 (0.15, 0.88)**
Urban Resident / Other	1.24 (0.71, 2.17)	1.17 (0.65, 2.1)	0.64 (0.32, 1.29)	0.62 (0.3, 1.26)	0.76 (0.23, 2.5)	0.56 (0.21, 1.51)
New Rural Coop (NCMS)	0.91 (0.59, 1.39)	0.86 (0.54, 1.37)	**0.45 (0.25, 0.82)**	**0.44 (0.24, 0.79)**	0.52 (0.22, 1.23)	0.61 (0.27, 1.36)
**United States**						
**Education Level**						
< High School (Ref)	-	-	-	-	-	-
High School / GED	0.94 (0.71, 1.24)	0.83 (0.62, 1.11)	0.91 (0.6, 1.38)	0.92 (0.61, 1.38)	0.8 (0.65, 1)	0.81 (0.63, 1.03)
> High School	0.96 (0.72, 1.28)	0.89 (0.65, 1.21)	0.92 (0.67, 1.26)	0.99 (0.71, 1.37)	0.93 (0.72, 1.2)	0.92 (0.7, 1.22)
**Family Income (PIR)**						
Quartile 1 (Lowest) (Ref)	-	-	-	-	-	-
Quartile 2	1.11 (0.83, 1.48)	1.11 (0.83, 1.49)	1.3 (0.94, 1.8)	1.19 (0.86, 1.67)	**1.3 (1.01, 1.66)**	**1.4 (1.09, 1.81)**
Quartile 3	1.12 (0.84, 1.5)	1.14 (0.84, 1.54)	1.17 (0.74, 1.86)	1.11 (0.71, 1.73)	1.21 (0.91, 1.6)	1.3 (0.97, 1.74)
Quartile 4 (Highest)	1.17 (0.8, 1.7)	1.15 (0.78, 1.68)	1.19 (0.75, 1.89)	1 (0.64, 1.57)	1.37 (0.98, 1.93)	**1.4 (1.02, 1.92)**

**Health Insurance Status**						
Uninsured (Ref)	-	-	-	-	-	-
Public Insurance	**1.91 (1.39, 2.64)**	**1.83 (1.32, 2.53)**	**1.84 (1.32, 2.58)**	**1.92 (1.39, 2.65)**	**1.44 (1.09, 1.9)**	**1.49 (1.1, 2.01)**
Private Insurance	**1.56 (1.16, 2.1)**	**1.57 (1.16, 2.12)**	**1.85 (1.22, 2.81)**	**1.94 (1.29, 2.91)**	1.3 (0.92, 1.86)	1.2 (0.82, 1.75)
**United Kingdom**						
**Education Level**						
Primary or below (Ref)	-	-	-	-	-	-
Middle/High School	1.1 (0.85, 1.42)	1.07 (0.83, 1.38)	**0.66 (0.47, 0.95)**	**0.69 (0.49, 0.97)**	1.06 (0.81, 1.4)	1.03 (0.76, 1.38)
College or above	**1.76 (1.13, 2.75)**	**1.86 (1.15, 3)**	0.73 (0.43, 1.23)	0.66 (0.4, 1.1)	0.77 (0.51, 1.16)	0.67 (0.42, 1.06)
**Total Household Wealth**						
Quartile 1 (Lowest) (Ref)	-	-	-	-	-	-
Quartile 2	1.28 (0.89, 1.83)	1.39 (0.97, 2.01)	1.36 (0.8, 2.31)	1.53 (0.93, 2.51)	1.38 (0.97, 1.97)	1.36 (0.91, 2.05)
Quartile 3	1.08 (0.75, 1.57)	1.25 (0.87, 1.79)	1.36 (0.82, 2.27)	1.62 (0.99, 2.65)	1.32 (0.92, 1.89)	1.38 (0.92, 2.08)
Quartile 4 (Highest)	0.92 (0.63, 1.34)	0.99 (0.68, 1.44)	1.1 (0.66, 1.84)	1.58 (0.96, 2.61)	**1.46 (1.02, 2.08)**	1.45 (0.95, 2.2)
**Private Health Insurance**						
No (Public Only) (Ref)	-	-	-	-	-	-
Yes	1.14 (0.76, 1.72)	1.15 (0.75, 1.76)	1.04 (0.56, 1.92)	0.85 (0.48, 1.51)	1.31 0.86, 2)	1.19 (0.73, 1.91)

Note: Data presentation: Data are presented as adjusted Odds Ratios (aOR) with 95% Confidence Intervals (CI). Boldface indicates statistical significance at $P < 0.05$. Models: Estimates were derived from survey-weighted multivariable logistic regression models. Model Definitions: Main Model: Adjusted for age, gender, race/ethnicity (US/China), smoking status, alcohol consumption, and BMI (consistent with the primary analysis). +Year Adj.: Adjusted for all covariates in the Main Model plus indicator variables for survey cycles/waves (e.g., NHANES cycles, CHARLS waves, ELSA waves) to control for potential temporal trends. Missing Data: Missing data for covariates were imputed using MICE. Participants with missing data for the specific outcome variable were excluded (listwise deletion).

Supplementary Table S6 Derivation of analytic cohorts and participant exclusion flow due to missing physiological indicators

Panel A. Hypertension Management

	Baseline Population	Hypertension Analysis Cohort				
Country	Total Participants (N)	Excluded: Missing Biomarkers/Outcomes (%)*	Excluded due to age < 20 (N)	Excluded: Missing Weights (N)	Excluded: Non-Hypertensive (N)	Final Analytic Sample (HTN) N
China	57,417	14,672 (25.6%)	18	250	24,400	18,077
US	59,842	19,442 (32.5%)	10,830	0	17,043	12,527
UK	79,208	49,543 (62.5%)	0	1,680	12,535	15,450

Panel B. Diabetes Management

	Baseline Population	Diabetes Analysis Cohort				
Country	Total Participants (N)	Excluded: Missing Biomarkers/Outcomes (%)*	Excluded due to age < 20 (N)	Excluded: Missing Weights (N)	Excluded: Non-Diabetic (N)	Final Analytic Sample (DM) N
China	57,417	35,448 (61.7%)	67	320	18,061	3,521
US	59,842	21,291 (35.6%)	6,784	0	25,865	5,902
UK	79,208	58,097 (73.3%)	0	62	18,667	2,382

Note: * Missing Biomarkers/Outcomes includes participants with missing blood pressure (for hypertension) or HbA1c/fasting glucose (for diabetes) measurements. Exclusion Rates: The proportion of participants excluded due to missing biomarkers varied by country due to different survey designs (e.g., biomarker collection was conducted only in specific subsamples or waves in NHANES and UK-ELSA, see Methods). Final Analytic Sample: Represents the unweighted count of participants aged ≥20 with the specific condition and valid survey weights, included in the main regression analysis.

Supplementary Table S7 Step-by-step sample attrition and unweighted participant counts across the hypertension and diabetes care cascades

Condition	Country	Total Cases (N)	Diagnosed	Treated	Controlled
			n (% of Total Cases*)	n (% of Diagnosed*)	n (% of Treated*)
Hypertension	China	18,077	12,138 (69.4%)	8,777 (72.3%)	3,770 (43.0%)
	US	12,527	10,609 (84.7%)	8,152 (76.8%)	5,341 (65.5%)
	UK	15,450	11,430 (74.0%)	7,612 (67.4%)	4,481 (59.4%)
Diabetes	China	3,521	1,664 (50.1%)	1,020 (61.3%)	464 (45.5%)
	US	5,902	4,555 (77.2%)	3,912 (85.9%)	1,863 (47.6%)
	UK	2,382	1,729 (72.6%)	1,433 (83.1%)	652 (45.5%)

Note: Data presented are unweighted sample counts (N or n) and unweighted percentages. * The denominators for percentages in the care cascade were calculated based on the number of participants at the previous stage, excluding those with missing data for the specific outcome variable (listwise deletion). Therefore, the sample sizes presented here correspond to the analytical samples used in the regression models.

References

16. Zhao, Y.; Hu, Y.; Smith, J.P.; Strauss, J.; Yang, G. Cohort profile: The China Health and Retirement Longitudinal Study (CHARLS). *Int. J. Epidemiol.* **2014**, *43*, 61–68. https://doi.org/10.1093/ije/dys203.

17. Steptoe, A.; Breeze, E.; Banks, J.; Nazroo, J. Cohort profile: The English longitudinal study of ageing. *Int. J. Epidemiol.* **2013**, *42*, 1640–1648. https://doi.org/10.1093/ije/dys168.

35. Curtin, L.R.; Mohadjer, L.K.; Dohrmann, S.M.; Kruszon-Moran, D.; Mirel, L.B.; Carroll, M.D.; Hirsch, R.; Burt, V.L.; Johnson, C. L. National Health and Nutrition Examination Survey: sample design, 2007-2010. *Vital Health Stat 2*. **2013**,*160*, 1-23.

36. Johnson, C.L.; Dohrmann, S.M.; Burt, V.L.; Mohadjer, L.K. National health and nutrition examination survey: sample design, 2011-2014. *Vital Health Stat 2*. **2014**,*162*, 1-33.

37. Chen, T.C.; Clark, J.; Riddles, M.K.; Mohadjer, L.K.; Fakhouri, T.H.I. National Health and Nutrition Examination Survey, 2015-2018: Sample Design and Estimation Procedures. *Vital Health Stat 2.* **2020**, *184*, 1-35.

38. Mirel, L.B.; Mohadjer, L.K.; Dohrmann, S.M.; Clark, J.; Burt, V.L.; Johnson, C. L.; Curtin, L. R. National Health and Nutrition Examination Survey: estimation procedures, 2007-2010. *Vital Health Stat 2.* **2013**, *159*, 1-17.

39. Chen, T.C.; Parker, J.D.; Clark, J.; Shin, H.C.; Rammon, J.R.; Burt, V.L. National Health and Nutrition Examination Survey: Estimation Procedures, 2011-2014. *Vital Health Stat 2.* **2018**, *177*, 1-26.
